# Fatigue among adult survivors of childhood cancer: findings from the French Childhood Cancer Survivor Study

**DOI:** 10.1007/s11764-026-01995-6

**Published:** 2026-03-14

**Authors:** Médéa Locquet, Duc Hoan Vu, Rodrigue Allodji, Neige Journy, Chiraz El-Fayech, Charlotte Demoor-Goldschmidt, Laura Lenez, Cécile Thomas-Teinturier, Stéphanie Bolle, Nadia Haddy, Giao Vu-Bezin, Agnès Dumas, David Rajaonera, Nicolas Bougas, Jacques Grill, Christelle Dufour, Hélène Pacquement, Ibrahima Diallo, Jean Bernard Le-Provost, François Doz, Brice Fresneau, Florent De Vathaire

**Affiliations:** 1 Inserm Research Unit 1018 for Epidemiology and Health Population (CESP), Université Paris-Saclay, UVSQ, Gustave Roussy, Villejuif, France; 2 University of Namur, Namur, Belgium; 3 Gustave Roussy, Villejuif, France; 4 Centro de Protonterapia, Grupo QuironSalud, Madrid, Spain; 5 IRD, ISSPAM, SESSTIM (Economic and Social Sciences of Health and Medical Information Processing), INSERM, Aix Marseille UnivCALIPSO Team, Marseille, France; 6 UMR 1123, Université de Paris, ECEVE, INSERM (National Institute for Health and Medical Research), Paris, France; 7 Institut Curie, Paris, France; 8 Inserm Research Unit 1355 - Molecular Radiotherapy and Innovative Therapeutics - (RAMO-IT), Université Paris-Saclay, Gustave Roussy, Villejuif, France

**Keywords:** Childhood cancer survivors, Fatigue, Radiotherapy, Cancer survivorship care, Quality of life, Cancer therapy late effects

## Abstract

**Introduction:**

Fatigue is a prevalent and late effect among childhood cancer survivors (CCSs). Few large studies have comprehensively evaluated its prevalence and determinants. Identifying risk factors for fatigue and severe fatigue (SF) is crucial to improving survivorship care. This study assessed the prevalence of fatigue and SF, as well as demographic, clinical, and treatment-related predictors in the French Childhood Cancer Survivor Study (FCCSS).

**Methods:**

This cross-sectional analysis included adult 5-year CCSs diagnosed before age 21 (1946–2000) in the FCCSS cohort. Fatigue was measured using the French Multidimensional Fatigue Inventory (MFI-20); SF was defined as a total score > 60. Associations with sex, age, cancer type, treatment, and social deprivation were estimated using multivariable regressions. Analyses were stratified by central nervous system (CNS) *vs.* non-CNS tumors and compared to general population norms.

**Results:**

Among 3170 CCSs, fatigue scores exceeded population norms from age 30, with women affected earlier and more severely. General (10.0 ± 4.2) and physical fatigue (9.6 ± 4.2) were the most pronounced dimensions, increasing with age and peaking at ≥ 50 years. Higher fatigue scores were independently associated with female sex, obesity, older age, and CNS tumor history. SF affected 16% of CCSs, more frequently in women (*OR* = 1.30), obese survivors (*OR* = 2.40), CNS tumor survivors (*OR* = 3.27), hematologic tumor survivors (*OR* = 1.46), and radiotherapy exposure (*OR* = 1.46).

**Conclusion:**

Fatigue remains a common, persistent late effect in CCSs. Female sex, older age, CNS tumors, and radiotherapy were strong predictors, underscoring the needs for targeted screening and long-term management.

**Supplementary Information:**

The online version contains supplementary material available at https://doi.org/10.1007/s11764-026-01995-6.

## Introduction

Over past decades, the prognosis of childhood cancer has significantly improved, with a 5-year survival rate reaching over 80% due to advances in diagnostic techniques and multimodal treatments [1]. This results in a growing population of childhood cancer survivors (CCSs), current prevalence being estimated at 6 million worldwide [2]. However, the burden of cancer treatment late effects is high [3–5].

Among these late effects, cancer-related fatigue (CRF) has emerged as one of the most common and debilitating secondary pathologies in CCSs [6]. The National Comprehensive Cancer Network defines CRF as a “distressing, persistent, subjective sense of physical, emotional, and/or cognitive tiredness or exhaustion related to cancer or cancer treatment, not proportional to recent activity and interferes with usual functioning” [7]. Importantly, unlike fatigue in the general population, CRF typically persists despite rest and severely impacts professional, educational, and social trajectories [8].

From a public health perspective, fatigue substantially contributes to increasing disability-adjusted life years (DALYs) in CCSs [9], while also increasing long-term dependence on health services [10]. The economic burden is also induced by productivity losses, work absenteeism/incapacity, and reduced educational achievements, especially when fatigue begins early in adulthood [10].

CRF etiology is multifactorial and not yet comprehensively understood, involving a complex interplay of biological, psychological, and social determinants. Current evidence suggests that persistent low-grade systemic inflammation contributes to the pathogenesis of fatigue, with elevated circulating levels of pro-inflammatory cytokines, such as interleukin-6 (IL-6), tumor necrosis factor-alpha (TNF-α), and C-reactive protein (CRP), frequently reported in long-term survivors with high fatigue scores [11, 12]. Neuroendocrine dysfunction has also been identified as a potential mechanism underlying energy balance impairments in CCSs [13]. Impaired mitochondrial oxidative function has been proposed as an additional biological contributor, although these pathways require further validation [14].

In epidemiological studies, between 19 and 42% of adult CCSs experienced clinically significant fatigue [6]. However, prevalence estimates vary across studies, possibly reflecting methodological heterogeneity in fatigue assessment tools, diagnostic criteria, and survivor characteristics [6]. Fatigue may persist independently of mood disorders or sleep disturbances, suggesting that fatigue represents a distinct clinical entity in CCSs [6].

Despite its frequency and impact, fatigue remains underdiagnosed and undermanaged in clinical routine after childhood cancer treatments [15, 16]. Further studies are needed to assess the prevalence of fatigue and severe fatigue (SF) in childhood cancer survivors (CCSs) and to identify demographic, clinical, cancer-related, and treatment-related risk factors within a population of adult survivors of childhood cancer. In the present study, we focused on a single cohort, the large-scale French Childhood Cancer Survivor Study (FCCSS), which includes adult long-term survivors of cancers diagnosed during childhood and adolescence. Such a study may help identify clinical and treatment-related risk factors and guide risk-adapted supportive care strategies.

## Methods

### Study population

The French Childhood Cancer Survivor Study (FCCSS) is a retrospective cohort of 7,670 individuals who survived at least 5 years after a solid cancer or lymphoma diagnosed before the age of 21 years, 1945 to 2000, across 5 French oncology centers. This study received approval from the French Data Protection Authority (CNIL, agreement No. 902287) and the Ethics Committee of the French National Institute of Health and Medical Research (INSERM). Written informed consent was obtained from all participants or legal representatives, in accordance with national and international ethical standards. This study was conducted in accordance with the principles of the Declaration of Helsinki (1964) and its subsequent amendments, and the European General Data Protection Regulation (GDPR, EU Regulation 2016/679).

### Fatigue assessment

Fatigue was measured using the French version of the Multidimensional Fatigue Inventory (MFI-20) [17], validated in the French general population. MFI-20 consists of 20 items assessing 5 domains: general fatigue, physical fatigue, reduced activity, reduced motivation, and mental fatigue. Each domain consists of 4 items, scored on a 5-point Likert scale (ranging from 1 to 5), with domain scores ranging from 4 to 20. Higher scores indicate greater fatigue. The global fatigue score ranges from 20 to 100. SF (SF) was defined as a total MFI-20 score > 60 based on literature findings suggesting clinical significance [17]. According to the scoring protocol, missing data were imputed with the mean of the 3 other items within the same dimension. The questionnaire was self-administered between 2005 and 2015. Participants with ≥ 1 missing response in any given domain were excluded.

### Cancer treatment exposure

Cancer treatment data were gathered from medical records using a standardized protocol. We considered treatment modalities such as radiotherapy (types of irradiation: head-and-neck, thoracic, abdomen, pelvis, leg and foot, arm and hand) and chemotherapy (types of agents: vinca alkaloids, alkylating agents, anthracyclines), both categorized as binary variables.

### Covariates

Covariates were selected based on their clinical relevance and prior literature findings [18, 19], including sex, age at cancer diagnosis, and age at fatigue assessment. The latter was categorized into age groups (< 30, 30–39, 40–49, ≥ 50 years) to account for potential aging-related variations in fatigue. Obesity (yes/no, defined as body mass index ≥ 30 kg/m^2^) was also reported. Covariates were the calendar year of cancer diagnosis, used as a proxy for treatment era and associated changes in therapeutic intensity and modalities, and the type of childhood cancer, classified according to the International Classification of Childhood Cancer (ICCC) [20]. The incidence of secondary pathologies during post-treatment follow-up, up to the response to the MFI-20 questionnaire (2005), was analyzed: second primary cancers, cardiovascular diseases, cerebrovascular diseases, renal diseases, and type II diabetes mellitus (yes/no). Socioeconomic status was considered, using the French Social Deprivation Index (SDI) [21]a validated ecological indicator derived from national data that reflects material and social deprivation at the municipal level. This index was categorized into tertiles to ensure sufficient group sizes and further statistical power: the highest tertile corresponds to a socially disadvantaged population.

### Statistical analysis

Descriptive statistics were applied to characterize the population. Continuous variables were summarized using mean ± standard deviation (SD). Categorical variables were described using absolute (*n*) and relative (%) frequencies. The distribution of continuous MFI-20 scores was assessed graphically. Skewed MFI-20 scores were log-transformed to allow parametric analyses. Comparisons of continuous variables across groups were conducted using Student’s *T*-test or one-way analysis of variance (ANOVA), as appropriate. Categorical variables were compared using *χ*^2^ tests.

Multiple linear regressions were applied to assess the association between clinical and sociodemographic variables and MFI-20 fatigue score, yielding a *β* coefficient and a 95% confidence interval (CI). Exponential *β* coefficient and 95% CI were further computed. Multivariable logistic regressions were fitted to estimate associations between clinical and sociodemographic characteristics and SF (Yes/No), yielding odds ratios (OR) and 95% CI. Models were adjusted for sex, obesity, age at childhood cancer diagnosis, age at questionnaire administration, year of cancer diagnosis, first cancer type, post-treatment secondary pathologies, radiotherapy, type of radiotherapy, chemotherapy, type of chemotherapy agents, and social deprivation index. Age at diagnosis and age at assessment were both included as they reflect distinct clinical dimensions (developmental stage at treatment and aging at survey), and multicollinearity was checked using variance inflation factors (VIF) and was found to be negligible (all VIF < 1.5). Stratified models were also used to evaluate potential heterogeneity in fatigue dimensions by cancer type (*i.e*., central nervous system (CNS) survivors *vs*. survivors of other cancers). As a sensitivity analysis, we further analyzed the subgroup of hematologic cancer survivors to assess associations with severe fatigue. Interaction effects between risk factors with fatigue (Wald test).

Comparison of fatigue scores between CCSs of the FCCSS and the general population was performed using sex- and age-specific thresholds defined by Schwarz et al. [22], corresponding to the 25th percentile of the German reference population, as no French normative data are available [22]. Accordingly, elevated fatigue was defined as MFI-20 general fatigue scores ≥ 9 for men aged 20–39 and ≥ 10 for men aged 40–59, and ≥ 10 for women aged 20–39 and ≥ 11 for women aged 40–59 [19, 23].

All statistical analyses were performed using SAS® (v.9.4, SAS Institute Inc.), and visualizations using R (v.3.6.3). Two-sided *p* < 0.05 was set as statistically significant.

## Results

### Participants characteristics

**Fig. 1:**
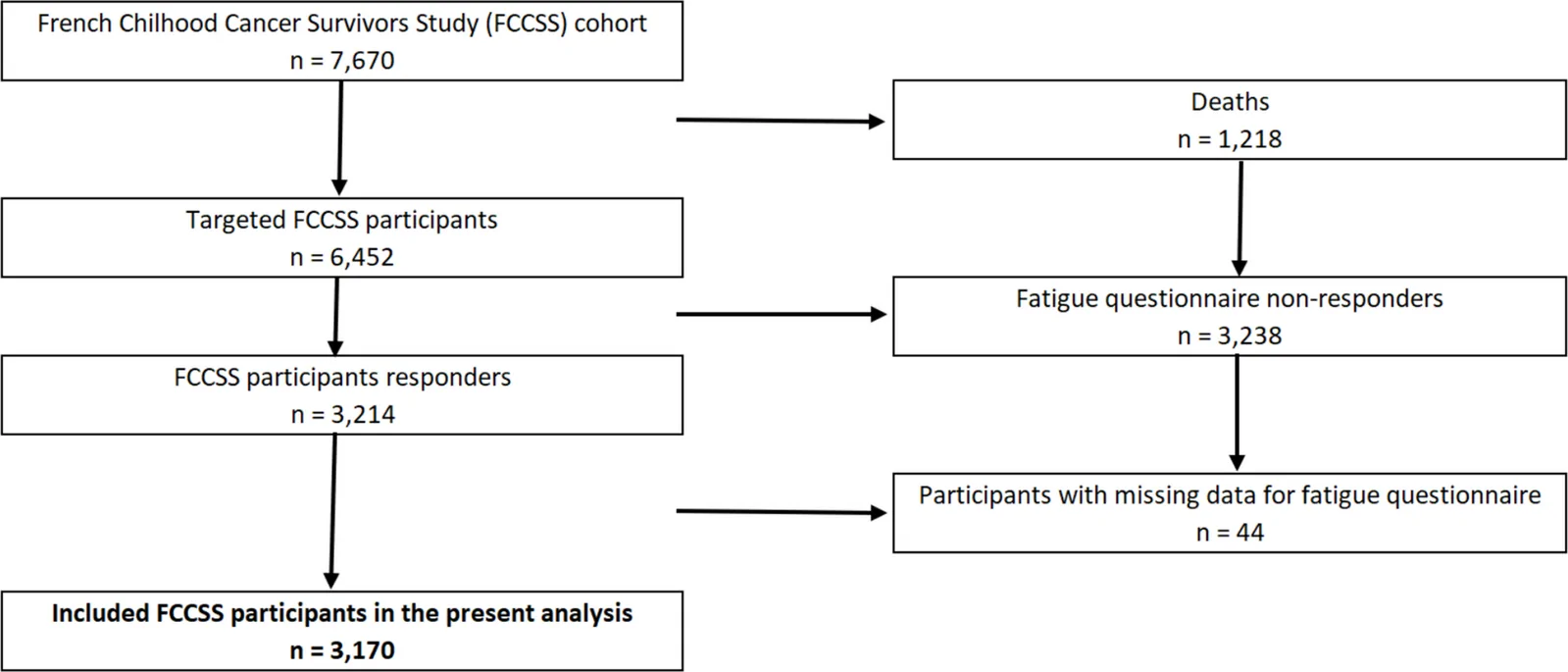
Flowchart of participant selection from the French Childhood Cancer Survivor Study (FCCSS) cohort

Among the 7,670 individuals included in the FCCSS cohort, 6,452 were alive at the time of the fatigue survey. Of these, 3,214 (49.8%) returned the self-administered questionnaire. After excluding 44 individuals (≥ 1 missing value in one dimension), a total of 3170 CCSs with complete data on MFI-20 were included in the present analysis. This corresponds to a final response rate of 41.3% (Fig. 1).

Compared with patients not included (i.e., non-responders and deceased patients), CCSs included showed distinct characteristics. Indeed, the distribution of the first cancer type differed significantly between included participants and those who refused or were lost to follow-up (*p*-value < 0.0001), indicating heterogeneity in inclusion across diagnostic groups. In addition, exposure to cancer treatments was significantly associated with inclusion status, with patients who received radiotherapy or chemotherapy more frequently represented among included survivors (*p* < 0.0001 and *p* < 0.01, respectively) (Supplementary Table 1).

Among the 3170 participants, 1,578 (49.8%) were men and 1,592 (50.2%) were women. The mean age at the time of fatigue questionnaire completion was 33.8 ± 8.7 years, and 27.4% had been diagnosed before age 1. Among CCSs, 19.9% experienced at least 1 secondary pathology, with second primary cancers being the most incident (10.6%). Radiotherapy was performed in 56.0% of CCSs, mainly thoracic (21.1%) and head-and-neck (19.9%) irradiation. Chemotherapy was administered to 77.7%, most commonly with vinca alkaloids (63.0%) (Table 1).

**Table 1:** Characteristics of participants from the French Childhood Cancer Survivors Study (FCCSS) cohort included in the present analysis (*n*=3,170)

Characteristics	Mean ± SD or *n* (%)
Sex	
*Women*	1,578 (49.8)
*Men*	1,592 (50.2)
Obesity	
*Yes*	245 (7.7)
*No*	2,925 (92.3)
Age at childhood cancer diagnosis (years)	6.1 ± 5.3
*0–1*	869 (27.4)
*2–4*	659 (20.8)
*5–8*	572 (18.0)
*9–12*	533 (16.8)
*13–20*	537 (17.0)
Age at questionnaire administration (years)	33.8 ± 8.7
*< 30*	1,071 (33.8)
*30–39*	1,325 (41.8)
*40–49*	614 (19.4)
*≥ 50*	160 (5.0)
Year of cancer diagnosis	
*1945–1975*	732 (23.1)
*1976–1980*	569 (18.0)
*1981–1985*	700 (22.1)
*1986–1990*	438 (13.8)
*1991–1995*	369 (11.6)
*1996–2000*	362 (11.4)
First cancer type	
*Lymphomas*	577 (18.2)
*Central nervous system tumors*	333 (10.5)
*Neuroblastomas*	463 (14.6)
*Retinoblastomas*	142 (4.5)
*Renal tumors*	595 (18.8)
*Hepatic tumors*	34 (1.0)
*Malignant bone tumors*	297 (9.4)
*Soft-tissue tumors*	380 (12.0)
*Germ cells and gonadal tumors*	224 (7.1)
*Other carcinomas*	125 (3.9)
Post-treatment secondary pathologies	
*Second primary cancers*	337 (19.9)
*Cardiovascular complications*	138 (4.3)
*Cerebrovascular complications*	16 (0.5)
*Renal diseases*	74 (2.3)
*Type II diabetes mellitus*	58 (1.8)
*At least 1 incident secondary pathology*	270 (8.5)
Radiotherapy	
* Yes*	1,774 (56.0)
* No*	1,396 (44.0)
* Head-and-neck irradiation*	631 (19.9)
* Thoracic irradiation*	668 (21.1)
* Abdomen irradiation*	670 (21.1%)
* Pelvis irradiation*	137 (4.3)
* Leg and foot irradiation*	82 (2.6)
* Arm and hand irradiation*	7 (0.2)
Chemotherapy	
* Yes*	2,462 (77.7)
* No*	708 (22.3)
* Vinca alkaloids*	1,998 (63.0)
* Alkylation agents*	1,794 (56.6)
* Anthracyclines*	1,239 (39.1)
Social deprivation index	
*1*^*st*^ *tertile*	989 (31.2)
*2*^*nd*^ *tertile*	989 (31.2)
*3*^*rd*^ *tertile*	989 (31.2)
*Not available*	203 (6.4)

### Fatigue assessment

The mean overall MFI-20 score was 42.4 ± 15.6, with general (10.0 ± 4.2) and physical fatigue (9.6 ± 4.2) scoring highest, and reduced motivation lowest (7.1 ± 3.1) (Table 2). Women reported higher fatigue than men (43.7 *vs*. 41.1, *p* < 0.0001). Fatigue was greater in CCSs diagnosed at 9–12 years (43.8 ± 16.3, *p* = 0.0002) and in those aged ≥ 50 years at assessment (47.0 ± 17.8, all *p* < 0.05). CNS tumor survivors had the highest scores (52.8 ± 17.1 across all dimensions; *p* < 0.0001), whereas no significant differences were found by SDI. CCSs with secondary pathologies reported higher overall fatigue, with the highest scores observed in CCSs with cerebrovascular complications (53.2 ± 17, *p* = 0.008). Fatigue was also greater in CCSs who received after Head-and-neck (46.6 ± 17.2) or thoracic irradiation (45.4 ± 15.6) (all *p* < 0.0001). No significant difference in fatigue across CCSs by chemotherapy agent class was observed.

**Table 2:** Multidimensional Fatigue Inventory (MFI-20) total and dimension scoring of the whole sample of childhood cancer survivors (CCSs), and according to sociodemographic and clinical characteristics (*n*=3,170)

	MFI-20 overall (/100)	General fatigue (/20)	Mental fatigue (/20)	Physical fatigue (/20)	Reduced activity (/20)	Reduced motivation (/20)
	Mean ± SD	Mean ± SD	Mean ± SD	Mean ± SD	Mean ± SD	Mean ± SD
Score, *mean ± standard deviation (SD)*	42.4 ± 15.6	10.0 ± 4.2	8.1 ± 3.7	9.1 ± 4.4	8.1 ± 4.0	7.1 ± 3.1
Sex						
*Women*	41.1 ± 15.2	9.2 ± 4.0	8.1 ± 3.6	8.5 ± 4.2	8.1 ± 3.9	7.1 ± 3.1
*Men*	43.7 ± 15.9	10.7 ± 4.2	8.0 ± 3.8	9.7 ± 4.4	8.2 ± 4.1	7.2 ± 3.2
*p*-value	< 0.0001	< 0.0001	0.46	< 0.0001	0.59	0.58
Obesity						
*Yes*	48.5 ± 17.4	11.3 ± 4.3	9.3 ± 4.2	10.9 ± 4.7	9.0 ± 4.4	8.1 ± 3.6
*No*	41.7 ± 15.2	9.8 ± 4.1	8.0 ± 3.6	8.9 ± 4.3	7.9 ± 8.2	7.0 ± 3.1
*p*-value	< 0.0001	< 0.0001	< 0.0001	< 0.0001	0.002	< 0.0001
Age at childhood cancer diagnosis (years)						
*0–1*	40.4 ± 14.9	9.4 ± 4.0	7.8 ± 3.5	8.6 ± 4.3	7.8 ± 3.8	6.8 ± 2.9
*2–4*	43.2 ± 15.4	10.2 ± 4.2	8.2 ± 3.7	9.3 ± 4.4	8.3 ± 4.0	7.2 ± 3.1
*5–8*	42.7 ± 15.9	10.1 ± 4.2	8.2 ± 3.9	9.1 ± 4.3	8.2 ± 4.0	7.1 ± 3.1
*9–12*	43.8 ± 16.3	10.3 ± 4.3	8.2 ± 3.8	9.5 ± 4.5	8.2 ± 4.1	7.4 ± 3.5
*13–20*	43.1 ± 15.6	10.2 ± 4.0	8.1 ± 3.7	9.4 ± 4.4	8.1 ± 4.0	7.3 ± 3.1
*p*-value	0.0002	< 0.0001	0.215	0.0004	0.10	0.0004
Age at questionnaire administration (years)						
*< 30*	40.5 ± 14.3	9.3 ± 3.9	8.0 ± 3.5	8.3 ± 4.0	8.1 ± 3.8	6.7 ± 2.9
*30–39*	42.6 ± 15.5	10.2 ± 4.1	8.0 ± 3.7	9.2 ± 4.4	8.1 ± 4.0	7.1 ± 3.1
*40–49*	44.3 ± 16.8	10.6 ± 4.3	8.2 ± 3.9	10.0 ± 4.6	8.1 ± 4.2	7.5 ± 3.3
*≥50*	47.0 ± 17.8	10.9 ± 4.4	9.0 ± 4.2	10.5 ± 4.6	8.4 ± 4.3	8.3 ± 3.8
*p*-value	< 0.0001	< 0.0001	0.02	< 0.0001	0.85	< 0.0001
Year of cancer diagnosis						
*1945–1975*	43.1 ± 16.3	10.3 ± 4.3	8.0 ± 3.8	9.8 ± 4.5	7.8 ± 4.1	7.3 ± 4.3
*1976–1980*	43.9 ± 16.5	10.4 ± 4.3	8.3 ± 3.9	9.7 ± 4.6	8.1 ± 4.1	7.4 ± 3.4
*1981–1985*	41.6 ± 14.9	9.9 ± 4.1	7.9 ± 3.5	8.8 ± 4.3	8.1 ± 3.8	6.9 ± 3.0
*1986–1990*	41.5 ± 15.4	9.8 ± 4.1	7.9 ± 3.7	8.7 ± 4.2	8.1 ± 3.8	7.0 ± 3.0
*1991–1995*	41.9 ± 15.2	9.8 ± 4.1	8.1 ± 3.7	8.8 ± 4.1	8.3 ± 4.0	6.9 ± 2.8
*1996–2000*	42.0 ± 14.4	9.5 ± 3.8	8.4 ± 3.6	8.4 ± 4.0	8.6 ± 4.0	7.1 ± 3.1
*p*-value	0.06	0.009	0.29	< 0.0001	0.03	0.02
First cancer type						
*Lymphomas*	42.0 ± 15.7	9.9 ± 4.2	8.1 ± 3.8	8.8 ± 4.4	8.1 ± 3.9	7.1 ± 3.2
*Central nervous system tumors*	52.8 ± 17.1	11.6 ± 4.1	10.3 ± 4.2	11.2 ± 4.6	10.6 ± 4.6	9.1 ± 3.7
*Neuroblastomas*	40.8 ± 14.9	9.6 ± 4.1	7.8 ± 3.6	8.7 ± 4.3	8.0 ± 3.9	6.8 ± 2.8
*Retinoblastomas*	39.3 ± 13.7	9.1 ± 3.9	7.8 ± 3.3	7.8 ± 3.6	7.7 ± 3.5	6.9 ± 3.0
*Renal tumors*	41.1 ± 14.6	10.0 ± 4.1	7.8 ± 3.6	9.1 ± 4.3	7.5 ± 3.6	6.7 ± 3.0
*Hepatic tumors*	38.4 ± 13.5	9.1 ± 4.2	7.4 ± 3.2	8.3 ± 4.3	7.0 ± 2.8	6.6 ± 2.8
*Malignant bone tumors*	42.7 ± 14.9	10.2 ± 4.1	7.8 ± 3.4	9.9 ± 4.2	7.9 ± 4.0	6.9 ± 3.1
*Soft-issue tumors*	40.4 ± 14.5	9.6 ± 4.1	7.6 ± 3.3	8.7 ± 4.2	7.7 ± 3.8	6.8 ± 2.9
*Germ cells and gonadal tumors*	40.2 ± 14.7	9.5 ± 4.2	7.8 ± 3.3	8.1 ± 4.1	7.8 ± 3.9	6.9 ± 2.7
*Other carcinomas*	43.5 ± 16.0	10.5 ± 4.1	8.2 ± 3.7	9.0 ± 4.2	8.3 ± 4.0	7.5 ± 3.2
*p*-value	< 0.0001	< 0.0001	< 0.0001	<0.0001	< 0.0001	< 0.0001
Post-treatment secondary pathologies						
*Second primary cancers*	46.1 ± 16.0	11.0 ± 4.1	8.6 ± 3.8	10.6 ± 4.6	8.2 ± 4.2	7.6 ± 3.4
*Cardiovascular complications*	45.3 ± 15.6	11.1 ± 4.2	8.2 ± 3.6	10.8 ± 4.9	8.1 ± 4.4	7.2 ± 3.0
*Cerebrovascular complications*	53.2 ± 17.3	12.1 ± 4.5	10.5 ± 3.4	11.7 ± 4.0	9.8 ± 4.1	9.1 ± 4.0
*Renal diseases*	46.8 ± 16.5	11.1 ± 4.2	8.9 ± 4.3	11.2 ± 4.7	8.0 ± 3.9	7.6 ± 3.1
*Type II diabetes mellitus*	47.4 ± 16.0	10.8 ± 4.3	9.0 ± 4.2	10.9 ± 4.3	8.8 ± 3.9	7.9 ± 3.2
*At least 1 incident secondary pathology*	46.3 ± 16.1	11.0 ± 4.2	8.6 ± 4.0	10.9 ± 4.7	8.3 ± 4.1	7.5 ± 3.2
*p-value*	0.06	0.46	0.008	0.23	0.05	0.02
Radiotherapy						
*Yes*	44.2 ± 15.1	10.3 ± 4.2	8.4 ± 3.8	9.8 ± 4.5	8.3 ± 4.1	7.3 ± 3.3
*No*	39.7 ± 14.2	9.4 ± 4.0	7.6 ± 3.4	8.2 ± 4.0	7.8 ± 3.8	6.7 ± 2.8
*p*-value	< 0.0001	< 0.0001	< 0.0001	< 0.0001	0.005	< 0.0001
Type of radiotherapy						
*Head-and-neck irradiation*	46.6 ± 17.2	10.5 ± 4.2	9.2 ± 4.1	10.0 ± 4.6	9.1 ± 4.4	8.1 ± 3.5
*Thoracic irradiation*	45.4 ± 15.6	10.7 ± 4.1	8.6 ± 3.8	10.1 ± 4.4	8.5 ± 4.1	7.5 ± 3.2
*Abdomen irradiation*	44.2 ± 15.3	10.6 ± 4.2	8.3 ± 3.8	10.0 ± 4.4	8.0 ± 3.9	7.3 ± 3.1
*Pelvis irradiation*	42.6 ± 14.4	10.2 ± 4.1	8.1 ± 3.4	10.1 ± 4.5	7.4 ± 3.5	6.7 ± 2.8
*Leg and foot irradiation*	43.1 ± 15.4	10.3 ± 4.1	8.0 ± 3.5	10.0 ± 4.3	7.5 ± 3.9	7.3 ± 3.4
*Arm and hand irradiation*	34.1 ± 10.7	8.4 ± 4.0	6.4 ± 1.8	7.6 ± 3.1	6.6 ± 2.8	5.1 ± 0.9
*p-value*	< 0.0001	0.52	< 0.0001	0.80	< 0.0001	< 0.0001
Chemotherapy						
*Yes*	41.8 ± 15.0	9.8 ± 4.1	8.0 ± 3.6	9.0 ± 4.3	8.0 ± 3.9	6.9 ± 3.0
*No*	43.6 ± 16.9	10.2 ± 4.8	8.4 ± 3.6	9.3 ± 4.4	8.4 ± 4.2	7.4 ± 3.4
*p*-value	<0.0001	0.05	0.02	0.20	0.03	0.0005
Type of chemotherapy agents						
*Vinca alkaloids*	41.6 ± 14.6	9.8 ± 4.1	7.9 ± 3.6	8.9 ± 4.3	8.0 ± 3.8	7.0 ± 3.0
*Alkylation agents*	42.1 ± 15.2	9.8 ± 4.2	8.1 ± 3.7	9.0 ± 4.3	8.1 ± 3.9	7.0 ± 3.1
*Anthracyclines*	41.4 ± 14.8	9.8 ± 4.1	7.9 ± 3.6	8.9 ± 4.3	7.9 ± 3.8	6.9 ± 3.0
*p-value*	0.39	0.99	0.17	0.73	0.36	0.60
Social deprivation index						
*1*^*st*^ *tertile*	43.2 ± 16.4	10.1 ± 4.3	8.3 ± 3.8	9.3 ± 4.4	8.2 ± 4.1	7.4 ± 3.4
*2*^*nd*^ *tertile*	42.0 ± 15.2	10.0 ± 4.1	8.0 ± 3.6	9.1 ± 4.4	8.1 ± 3.9	7.0 ± 3.0
*3*^*rd*^ *tertile*	41.9 ± 15.1	9.9 ± 4.0	7.9 ± 3.6	8.9 ± 4.3	8.1 ± 3.9	7.0 ± 3.0
*Not available*	43.5 ± 15.4	10.0 ± 4.2	8.5 ± 3.7	9.6 ± 4.4	8.2 ± 4.0	7.1 ± 3.1
*p*-value	0.15	0.80	0.03	0.14	0.89	0.03

*CI* confidence interval

### Factors associated with fatigue

Table 3 reports multivariate associations between demographic, socioeconomic, and clinical characteristics and overall fatigue scores, with back-transformed coefficients on the exponential scale (regression coefficients for the log of scores: Supplementary Table 2).

**Table 3:** Associations between sociodemographic and clinical characteristics and MFI-20 fatigue score in childhood cancer survivors (CCSs) using multivariable linear regressions

	MFI-20 overall Adjusted^b^ β [95%CI]	General fatigue Adjusted β^b^ [95%CI]	Mental fatigue Adjusted^b^ β [95%CI]	Physical fatigue Adjusted^b^ β [95%CI]	Reduced activity Adjusted^b^ β [95%CI]	Reduced motivation
						Adjusted^b^ β [95%CI]
Sex						
*Men*	Reference	Reference	Reference	Reference	Reference	Reference
*Women*	1.06 [1.03–1.09]^a^	1.19 [1.15–1.23]^a^	0.98 [0.95–1.01]	1.15 [1.11–1.19]^a^	1.01 [0.98–1.03]	1.01 [0.98–1.03]
Obesity						
*No*	Reference	Reference	Reference	Reference	Reference	Reference
*Yes*	1.15 [1.07–1.25]^a^	1.17 [1.07–1.28]^a^	1.14 [1.04–1.25]^a^	1.27 [1.15–1.35]^a^	1.08 [1.00–1.21]	1.08 [1.00–1.17]^a^
Age at childhood cancer diagnosis (*years*)						
*0–1*	Reference	Reference	Reference	Reference	Reference	Reference
*2–4*	1.04 [1.00–1.08]^a^	1.05 [1.01–1.11]	1.03 [0.98–1.08]	1.04 [0.99–1.09]	1.04 [0.99–1.09]	1.04 [1.00–1.08]^a^
*5–8*	0.98 [0.94–1.03]	1.01 [0.96–1.06]	0.97 [0.92–1.03]	0.99 [0.93–1.05]	0.96 [0.90–1.02]	0.98 [0.94–1.03]
*9–12*	0.99 [0.94–1.04]	1.01 [0.95–1.07]	0.97 [0.91–1.03]	1.02 [0.95–1.09]	0.94 [0.88–1.01]	0.99 [0.94–1.04]
*13–20*	0.98 [0.92–1.03]	1.00 [0.93–1.07]	0.96 [0.90–1.03]	1.02 [0.95–1.12]	0.91 [0.83–0.98]	0.98 [0.92–1.03]
Age at questionnaire administration (*years*)						
*<30*	Reference	Reference	Reference	Reference	Reference	Reference
*30–39*	1.07 [1.04–1.13]^a^	1.13 [1.06–1.19]^a^	1.02 [0.97–1.07]	1.08 [1.03–1.16]^a^	1.07 [1.02–1.14]^a^	1.08 [1.04–1.16]^a^
*40–49*	1.16 [1.08–1.23]	0.19 [0.110.27]^a^	1.07 [1.00–1.16]	1.17 [1.08–1.30]^a^	1.15 [1.05–1.26]^a^	1.19 [1.12–1.88]^a^
*≥ 50*	1.23 [1.13–1.38]^a^	1.25 [1.09–1.42]^a^	0.16 [0.06–0.23]^a^	1.22 [1.07–1.40]^a^	1.25 [1.09–1.43]^a^	1.32 [1.19–1.52]^a^
Year of cancer diagnosis						
*1945–1975*	Reference	Reference	Reference	Reference	Reference	Reference
*1976–1980*	1.06 [1.01–1.12]^a^	1.05 [0.99–1.12]	1.06 [1.01–1.14]	1.03 [0.97–1.09]	1.09 [1.03–1.17]^a^	1.06 [1.01–1.13]^a^
*1981–1985*	1.05 [1.00–1.12]	1.05 [0.99–1.13]	1.04 [0.98–1.13]	0.97 [0.90–1.04]	1.15 [1.07–1.25]^a^	1.04 [0.98–1.12] 1.05 [0.98–1.13]
*1986–1990*	1.04 [0.98–1.09]	1.04 [0.97–1.13]	1.04 [0.97–1.13]	0.95 [0.89–1.03]	1.16 [1.06–1.26]^a^	
*1991–1995*	1.05 [0.99–1.14]	1.04 [0.96–1.14]	1.08 [1.00–1.19]	0.96 [0.89–1.05]	1.21 [1.08–1.32]^a^	1.07 [0.99–1.16]
*1996–2000*	1.09 [1.02–1.19]^a^	1.05 [0.96–1.16]	1.13 [1.03–1.25]^a^	0.95 [0.87–1.05]	1.31 [1.17–1.51]^a^	1.13 [1.02–1.23]^a^
First cancer type						
*Lymphomas*	Reference	Reference	Reference	Reference	Reference	Reference
*Central nervous system tumors*	1.30 [1.23–1.38]^a^	1.19 [1.12–1.27]^a^	1.31 [1.25–1.42]^a^	1.35 [1.25–1.46]^a^	1.38 [1.25–1.52]^a^	1.32 [1.23–1.42]^a^
*Neuroblastomas*	0.99 [0.94–1.04]	0.99 [0.92–1.05]	0.98 [0.92–1.05]	1.00 [0.93–1.07]	1.00 [0.93–1.06]	0.99 [0.93–1.05]
*Retinoblastomas*	0.96 [0.89–1.03]	0.94 [0.85–1.03]	0.97 [0.89–1.06]	0.93 [0.83–1.02]	0.97 [0.87–1.06]	1.00 [0.92–1.07]
*Renal tumors*	0.98 [0.93–1.03]	1.01 [0.95–1.06]	0.96 [0.91–1.02]	1.02 [0.96–1.08]	0.94 [0.88–1.00]	0.95 [0.90–1.00]
*Hepatic tumors*	0.96 [0.84–1.08]	0.95 [0.80–1.12]	0.94 [0.79–1.09]	1.00 [0.81–1.20]	0.88 [0.73–1.06]	1.00 [0.86–1.16]
*Malignant bone tumors*	1.02 [0.97–1.07]	1.02 [0.96–1.08]	0.98 [0.92–1.04]	1.15 [1.06–1.23]a	0.98 [0.91–1.04]	0.96 [0.91–1.02]
*Soft-tissue tumors*	0.96 [0.91–1.01]	0.96 [0.90–1.01]	0.96 [0.90–1.02]	0.98 [0.92–1.04]	0.95 [0.89–1.01]	0.96 [0.91–1.01]
*Germ cells and gonadal tumors*	0.96 [0.90–1.01]	0.94 [0.87–1.01]	0.99 [0.92–1.06]	0.90 [0.83–0.97]^a^	0.98 [0.91–1.04]	0.99 [0.93–1.06]
*Other carcinoma*	1.02 [0.95–1.09]	1.02 [0.94–1.13]	1.02 [0.93–1.12]	1.00 [0.90–1.09]	1.04 [0.95–1.15]	1.04 [0.97–1.14]
Post-treatment secondary pathologies						
*None*	Reference	Reference	Reference	Reference	Reference	Reference
*Second primary cancers*	1.08 [1.04–1.13]^a^	1.08 [1.05–1.15]^a^	1.05 [0.94–1.17]^a^	1.10 [1.01–1.22]^a^	1.27 [0.91–1.60]	0.97 [0.92–1.08]^a^
*Cardiovascular complications*	1.07 [1.01–1.14]^a^	1.09 [1.04–1.15]^a^	1.12 [1.05–1.17]^a^	1.10 [0.99–1.23]	1.22 [0.97–1.36]	1.26 [1.00–1.58]
*Cerebrovascular complications*	1.23 [1.03–1.47]^a^	1.13 [1.04–1.21]^a^	1.08 [1.04–1.14]^a^	1.07 [1.01–1.13]^a^	1.16 [0.98–1.26]	1.02 [0.92–1.14]
*Renal diseases*	1.12 [1.02–1.20]^a^	1.20 [0.97–1.47]	1.03 [0.96–1.10]	1.14 [1.08–1.20]^a^	1.18 [0.96–1.26]	1.09 [0.99–1.22]
*Type II diabetes mellitus*	1.10 [1.01–1.21]^a^	1.12 [1.08–1.23]^a^	1.34 [1.08–1.66]^a^	1.18 [1.08–1.28]^a^	1.02 [0.98–1.08]	1.07 [1.01–1.12]^a^
*At least 1 incident secondary pathology*	1.08 [1.05–1.15]^a^	1.12 [1.05–1.17]^a^	1.07 [1.05–1.13]^a^	1.19 [1.13–1.26]^a^	1.04 [0.99–1.11]	1.07 [1.01–1.13]^a^
Radiotherapy						
*No*	Reference	Reference	Reference	Reference	Reference	Reference
*Yes*	1.06 [1.03–1.09]	1.05 [1.02–1.09]	1.07 [1.04–1.13]	1.13 [1.07–1.32]	1.02 [0.98–1.65]	1.04 [1.01–1.07]
Type of radiotherapy						
*None*	Reference	Reference	Reference	Reference	Reference	Reference
*Head-and-neck irradiation*	1.11 [1.08–1.15]^a^	0.81 [0.62–1.06]	1.02 [0.93–1.12]	1.02 [0.95–1.09]	1.12 [1.07–1.16]^a^	1.06 [1.02–1.10]^a^
*Thoracic irradiation*	1.08 [1.05–1.13]^a^	1.07 [1.03–1.10]^a^	0.82 [0.60–1.13]	0.99 [0.89–1.09]	1.08 [1.01–1.17]^a^	1.01 [0.97–1.05]
*Abdomen irradiation*	1.06 [1.03–1.09]^a^	1.08 [1.05–1.13]^a^	1.16 [1.12–1.19]^a^	0.85 [0.62–1.17]	1.07 [0.98–1.20]	0.93 [0.86–1.01]
*Pelvis irradiation*	1.01 [0.95–1.06]	1.07 [1.03–1.12]^a^	1.09 [1.05–1.13]^a^	1.10 [1.05–1.15]^a^	0.84 [0.77–1.20]	0.94 [0.85–1.04]
*Leg and foot irradiation*	1.01 [0.94–1.10]	1.01 [0.95–1.09]	1.05 [1.01–1.09]^a^	1.14 [1.09–1.18]^a^	1.15 [1.10–1.20]^a^	1.15 [1.12–1.20]^a^
*Arm and hand irradiation*	1.02 [0.97–1.04]	0.99 [0.94–1.03]	0.91 [0.86–1.11]	0.97 [0.88–1.10]	1.01 [0.94–1.14]	0.96 [0.87–1.05]
Chemotherapy						
*No*	Reference	Reference	Reference	Reference	Reference	Reference
*Yes*	1.03 [0.99–1.06]	1.02 [0.98–1.06]	1.01 [0.97–1.04]	1.04 [0.99–1.08]	1.02 [0.98–1.07]	1.03 [0.99–1.06]
Type of chemotherapy agents						
*None*	Reference	Reference	Reference	Reference	Reference	Reference
*Vinca alkaloids*	0.99 [0.97–1.01]	0.99 [0.97–1.03]	0.98 [0.95–1.01]	0.99 [0.96–1.03]	0.98 [0.94–1.01]	1.01 [0.98–1.03]
*Alkylation agents*	1.01 [0.99–1.03]	0.99 [0.97–1.02]	1.01 [0.98–1.03]	1.01 [0.98–1.04]	1.01 [0.98–1.05]	0.97 [0.94–0.99]
*Anthracyclines*	0.99 [0.96–1.02]	1.01 [0.97–1.03]	0.97 [0.94–0.99]	1.02 [0.98–1.05]	0.96 [0.93–0.99]	0.95 [0.91–0.99]
Social deprivation index						
*1*^*st*^ *tertile*	Reference	Reference	Reference	Reference	Reference	Reference
*2*^*nd*^ *tertile*	0.98 [0.95–1.01]	0.99 [0.95–1.03]	0.97 [0.93–1.03]	0.98 [0.94–1.02]	0.99 [0.95–1.03]	0.96 [0.92–1.00]^a^
*3*^*rd*^ *tertile*	0.99 [0.96–1.02]	1.00 [0.96–1.04]	0.97 [0.93–1.00]	0.98 [0.94–1.03]	1.00 [0.96–1.04]	0.97 [0.94–1.01]
*Not available*	1.02 [0.97–1.08]	1.01 [0.94–1.07]	1.03 [0.96–1.09]	1.05 [0.98–1.13]	1.02 [0.95–1.09]	0.99 [0.93–0.95]

*CI* confidence interval

^a^ *p* < 0.05

^b^Models adjusted for sex, obesity, age at childhood cancer diagnosis, age at questionnaire administration, year of cancer diagnosis, first cancer type, post-treatment secondary pathologies, radiotherapy, type of radiotherapy, chemotherapy, type of chemotherapy agents, and social deprivation index

Sex was significantly associated with fatigue, with women showing a relative increase of + 6% in total fatigue score, with higher levels of + 19% in general fatigue compared to men (*p* < 0.001).

Obesity was consistently associated with higher reported fatigue across most dimensions. Compared to non-obese CCSs, obese survivors had a relative increase of +15% in total fatigue, +17% in general fatigue, +14% in mental fatigue, +27% in physical fatigue, and +8% in reduced motivation (all *p* < 0.05).

Older age at questionnaire administration was also associated with higher fatigue scores. Compared to participants aged <30 years, those aged ≥50 years showed the strongest associations, with a relative increase of +23% in total fatigue, and of +25% in general fatigue, +32% in mental fatigue, +16% in physical fatigue, +22% in reduced activity, and +25% in reduced motivation (all *p* <0.05). 

Regarding the year of cancer diagnosis, CCSs diagnosed between 1996 and 2000 had significantly higher levels of + 13% in physical fatigue, + 7% in reduced motivation, and + 13% in total fatigue compared to those diagnosed in 1945–1975 (all *p* < 0.05). Among cancer types, CNS tumor survivors reported the highest fatigue burden across all dimensions. Compared to survivors of lymphomas (reference), CNS tumor survivors had a relative increase of + 30% in total fatigue, + 19% in general fatigue, + 31% in mental fatigue, + 35% in physical fatigue, + 38% in reduced activity, and + 32% in reduced motivation (all *p* < 0.001).

Post-treatment secondary pathologies were significantly associated with higher overall fatigue: compared to survivors without complications, those with cardiovascular conditions showed a + 7% increase, cerebrovascular complications a + 23% increase, renal diseases a + 12% increase, and type II diabetes a + 10% increase in overall fatigue scores (all *p* < 0.05). The type of radiotherapy was significantly associated with overall fatigue, with head-and-neck irradiation (+ 11%), thoracic irradiation (+ 8%), and abdominal irradiation (+ 6%) showing increased fatigue compared to survivors not receiving radiotherapy (all *p* < 0.05). Conversely, the different types of chemotherapy agents were not significantly associated with fatigue (all *p* > 0.05).

Finally, CCSs from the most deprived SDI tertile had higher reduced motivation scores, with a relative increase of + 4% compared to the least deprived group (*p* < 0.05). An interaction effect between the studied factors affected reported fatigue: the combination of female sex and younger age at cancer diagnosis was significantly associated with higher fatigue levels, as measured by the overall MFI-20 score (*p* = 0.03), suggesting a synergistic effect of these variables on fatigue.

### Factors associated with SF

SF was reported in 16% of CCSs. Table 4 describes the associations between sociodemographic and clinical characteristics and SF. Compared with men, women had a 27% higher odds of reporting SF (*p* < 0.05). Obese survivors also showed a 2.21-fold increase in the odds of reporting SF compared to non-obese CCSs. Age at assessment was strongly associated with SF in all age groups (all *p* < 0.05), particularly in the ≥ 50 group (OR = 4.40, 95% CI: [2.27–8.54]). Compared with the reference period (1946–1975), individuals diagnosed in 1976–1980, 1981–1985, 1986–1990, 1991–1995, and 1996–2000 showed progressively higher odds of reporting SF. The highest OR was observed in CCSs diagnosed in 1996–2000 (*OR* = 1.85; 95% CI: 1.02–3.36), followed by those diagnosed in 1976–1980 (*OR* = 1.50; 95% CI: 1.06–2.12). Primary cancer type was also significantly associated with SF: CNS tumor survivors showed more than threefold higher odds of SF compared to the reference (i.e., diagnosis of lymphoma). CCSs with at least one secondary pathology had 62% increased odds of SF (OR = 1.62, 95% CI: 1.18–2.21). Regarding treatment exposures, CCSs who received radiotherapy had a significantly higher odds of SF than those who were not exposed (*OR* = 1.42; 95% CI: 1.12–1.80). The type of radiotherapy was also strongly associated with SF: Head-and-neck irradiation was associated with the highest odds ratio (OR = 2.11, 95% CI: 1.69–2.62), followed by thoracic irradiation (OR = 1.34, 95% CI: 1.06–1.69). Abdominal, pelvic, and limb irradiation were not significantly associated. The effect of radiation therapy was similar in patients treated for a CNS tumor (Supplementary Table 3, OR = 1.38, 95% CI: 0.57–3.34) and for the other patients of FCCSS (Supplementary Table 4, OR = 1.42, 95% CI: 1.11–1.83), and mainly driven by radiation dose received to the brain (Supplementary Table 5), the one received by lungs and heart playing a non-significant role. No significant associations were observed between SF and chemotherapy, type of chemotherapy agent, or SDI, after adjustment for covariates.

**Table 4:** Associations between sociodemographic and clinical characteristics and the reporting of severe fatigue in CCSs of the FCCSS cohort

Characteristics	Adjusted^a^ OR [95%CI]
Sex	
*Men (reference)*	1.00
*Women*	1.28 [1.03–1.56]
Obesity	
*No (reference)*	1.00
*Yes*	2.40 [1.37–3.59]
Age at childhood cancer diagnosis (years)	
*0–1 (reference)*	1.00
*2–4*	1.16 [0.85–1.60]
*5–8*	0.86 [0.60–1.24]
*9–12*	0.81 [0.54–1.22]
*13–20*	0.67 [0.42–1.06]
Age at questionnaire administration (years)	
*< 30 (reference)*	1.00
*30–39*	1.72 [1.23–2.40]
*40–49*	3.07 [1.91–4.93]
*≥ 50*	4.40 [2.27–8.54]
Year of cancer diagnosis	
*1945–1975 (reference)*	1.00
*1976–1980*	1.50 [1.06–2.12]
*1981–1985*	1.33 [0.88–2.01]
*1986–1990*	1.52 [0.96–2.42]
*1991–1995*	1.69 [0.98–2.90]
*1996–2000*	1.85 [1.02–3.36]
First cancer type	
*Lymphomas (reference)*	1.00
*Central nervous system tumors*	3.05 [2.10–4.43]
*Neuroblastomas*	1.13 [0.73–1.73]
*Retinoblastomas*	0.62 [0.31–1.23]
*Renal tumors*	0.83 [0.56–1.22]
*Hepatic tumors*	1.21 [0.40–3.65]
*Malignant bone tumors*	1.14 [0.76–1.72]
*Soft-tissue tumors*	0.94 [0.63–1.39]
*Germ cells and gonadal tumors*	1.02 [0.63–1.64]
*Other carcinomas*	1.53 [0.89–2.66]
Post-treatment secondary pathologies	
*None (reference)*	1.00
*Second primary cancers*	1.30 [0.97–1.71]
*Cardiovascular complications*	0.65 [0.35–1.22]
*Cerebrovascular complications*	2.00 [0.56–6.77]
*Renal diseases*	1.47 [0.74–2.83]
*Type II diabetes mellitus*	1.11 [0.78–2.22]
*At least 1 incident secondary pathology*	1.62 [1.18–2.21]
Radiotherapy	
*No (reference)*	1.00
*Yes*	1.42 [1.12–1.80]
Type of radiotherapy	
*None (reference)*	1.00
*Head-and-neck irradiation*	2.11 [1.69–2.62]
*Thoracic irradiation*	1.34 [1.06–1.69]
*Abdomen irradiation*	1.16 [0.91–1.47]
*Pelvis irradiation*	0.77 [0.44–1.26]
*Leg and foot irradiation*	1.16 [0.61–2.05]
*Arm and hand irradiation*	0.30 [0.09–1.89]
Chemotherapy	
*No (reference)*	1.00
*Yes*	1.10 [0.84–1.44]
Type of chemotherapy agents	
*None (reference)*	1.00
*Vinca alkaloids*	0.85 [0.69–1.05]
*Alkylation agents*	1.06 [0.84–1.34]
*Anthracyclines*	0.96 [0.75–1.22]
Social deprivation index	
*1*^*st*^ *tertile (reference) (least deprived)*	1.00
*2*^*nd*^ *tertile*	0.80 [0.62–1.02]
*3*^*rd*^ *tertile (most deprived)*	0.81 [0.63–1.04]
*Not available*	1.02 [0.68–1.54]

*CCS* childhood cancer survivor; *FCCSS* French Childhood Cancer Survivors Study; *OR* odds ratio; *CI* confidence interval

^a^Models adjusted for sex, obesity, age at childhood cancer diagnosis, age at questionnaire administration, year of cancer diagnosis, first cancer type, post-treatment secondary pathologies, radiotherapy, type of radiotherapy, chemotherapy, type of chemotherapy agents, and social deprivation index

In a stratified multivariable logistic regression analysis, results differed between CNS tumor survivors and those with other cancer types (Supplementary Tables 2 and 3). Among CNS tumor survivors, none of the sociodemographic or clinical variables were explicitly associated with the risk of SF. In contrast, among non-CNS tumor survivors, women presented higher odds of SF (OR = 1.29, 95% CI: 1.03–1.61). Age at cancer diagnosis showed a protective effect in the 13–20-year group compared with the 0–1-year group (*OR* = 0.59, 95% CI: 0.35–0.99). Age at questionnaire administration was strongly associated with higher odds of SF across age groups: 30–39 years (*OR* = 2.16, 95% CI 1.49–3.13), 40–49 years (*OR* = 3.70, 95% CI: 2.20–6.22), and ≥ 50 years (*OR* = 4.71, 95% CI: 2.28–9.74), compared to non-CNS survivors aged < 30 years. Regarding cancer treatments, exposure to radiotherapy showed a significant 46% [1.14–1.87] higher odds of SF.

In the sensitivity analysis, hematological cancer survivors reported a higher odds of SF compared with other non-hematologic cancer survivors (adjusted OR 1.46, 95% CI 1.07–1.99).

### Fatigue comparison between CCSs and general population

Compared with the German general population, CCS reported higher general fatigue scores through age 59, particularly among women (mean 10.4 and 11.4 for ages ≤ 39 and 40–59 vs. 7.7 and 8.7 in the general population). The same trend was observed in men, though less marked (9.0 and 9.7 vs. 6.6 and 8.0, respectively). In both sexes aged ≥ 60, fatigue levels were comparable to those of the general population (Table 5).

**Table 5:** Comparisons of general fatigue scores using the MFI-20 in FCCSS and general population (German reference of [22]) [22] according to age groups^a^

Sex	Age group (*years*)	FCCSS mean (/20)	FCCSS 75th percentile (/20)	German general population mean (/20)	German general population 75th percentile (/20)	Interpretation
*Female*	≤ *39*	10.4	13	7.7	10	Higher fatigue level
*Female*	*40–59*	11.4	15	8.7	11	Higher fatigue level
*Female*	≥ *60*	11.6	13	10.8	13	Comparable to the general population
*Male*	≤ *39*	9.0	11	6.6	8	Higher fatigue level
*Male*	*40–59*	9.7	13	8.0	10	Higher fatigue level
*Male*	≥ *60*	10.6	15	10.1	13	Comparable to the general population

^a^French scoring guide[23] summarizing German epidemiological norms (derived from Schwarz et al., 2003[22], and Singer et al*.*, 2011[19]) for “general fatigue,” corresponding to the 25th percentile

## Discussion

### Findings

The main findings highlighted the high prevalence and multifactorial nature of fatigue in CCSs, with significant determinants such as sex, age, obesity, post-treatment secondary pathologies, radiotherapy type, and cancer type. Indeed, female sex, older age at assessment, obesity, and radiotherapy (especially head-and-neck irradiation) were consistently associated with increased fatigue levels or SF, suggesting that these characteristics may identify high-risk subgroups. Importantly, CNS tumor survivors showed the highest overall fatigue burden, independent of sociodemographic or treatment-related factors, underscoring this specific CCS population vulnerability. Our results were consistent with prior research identifying female sex, increasing age, obesity, and CNS tumor history as associated with higher fatigue burden in CCSs [24–26].

Female survivors showed a significantly higher risk of fatigue and SF, with 27% increased odds compared to males. Another Swiss study of adult CCSs was in line with our findings: female sex was associated with a nearly twofold risk of higher fatigue in adult CCSs [24]. These sex differences may reflect underlying biological mechanisms, such as heightened systemic inflammation and hormonal influences that predispose women to persistent fatigue, both in cancer survivors and in the general population [27]. In addition, it is known that CCSs are at elevated risk of impaired fertility and adverse reproductive outcomes (*e.g*., premature ovarian failure, miscarriage, preterm birth) because of cancer therapies [28]. Hence, motherhood could further contribute to long-term fatigue through hormonal, physical, and caregiving burdens, particularly among those who have experienced fertility or reproductive health challenges. Finally, women seem to report psychosocial stressors more frequently, which could exacerbate fatigue perception and severity[29]. These findings underscore the need to implement sex-specific fatigue awareness in clinical settings and individualized supportive care strategies.

Obesity was independently associated with a higher odd of SF, suggesting that metabolic dysregulation [30] may play a key role in long-term fatigue among CCSs, supporting the inclusion of metabolic health monitoring and lifestyle interventions in supportive care to mitigate fatigue risk in CCSs [18]. To our knowledge, no prior study has highlighted such differences.

A temporal trend in the risk of fatigue burden and SF was observed across cancer diagnosis periods, with survivors treated in more recent decades showing progressively higher odds of reporting fatigue. This pattern may reflect the long-term consequences of evolving treatment strategies, including more intensive or multimodal treatment. Although therapeutic advances contributed to improved survival, changes in the profiles of cancer survivors, such as prolonged follow-up durations and heightened awareness of late effects, may have influenced fatigue symptom reporting and detection or screening [31].

Primary cancer type also emerged as a significant determinant of fatigue and SF, with CNS tumor survivors reporting a significantly higher risk compared to other cancer groups. This exacerbated vulnerability may reflect the cumulative burden of the tumor itself, its neuroanatomical location, and the neurotoxic effects of CNS therapies such as cranial irradiation, in line with what was suggested in other studies [6, 32]. Tumor-specific CCSs trajectories and adapted supportive care should then be adapted accordingly. In addition, the sensitivity analysis suggested that hematological cancer survivors had a higher odd of SF (adjusted *OR* = 1.46). This finding aligns with previous studies pooled in a systematic review, reporting that fatigue represents a frequent and persistent late effect among CCSs of hematological malignancies, including Hodgkin lymphoma survivors [33]. This association may be related to a distinct profile of therapeutic exposures and late effects in hematologic cancer. For instance, systemic chemotherapy and, historically, thoracic irradiation in lymphoma survivors may contribute to cardiopulmonary or endocrine complications, chronic inflammation, sleep disturbances, and reduced physical capacity, all of which have been recognized as key pathways underlying persistent cancer-related fatigue in CCSs [34]. Together, these findings suggested that the burden is of huge importance in hematological cancer survivors as a subgroup warranting specific surveillance and supportive care in survivorship follow-up [11, 35].

Post-treatment secondary pathologies, particularly cerebrovascular, renal, and cardiometabolic complications, were strongly associated with SF in CCSs, consistent with prior evidence linking late morbidity to chronic fatigue. In the Dutch DCCSS LATER cohort, the incidence of ≥ 1 comorbidity doubled the fatigue risk, underscoring multimorbidity as a key driver of fatigue alongside treatment late effects [36].

Radiotherapy exposure was significantly associated with strongly increased odds of fatigue and SF in CCSs, in line with the known late effects of radiation-induced side effects on multiple physiological systems [37]. Another study found that CCSs with a history of radiotherapy were more likely to be fatigued [26] (OR = 1.7 [1.3–2.3] in Mulrooney et al*. vs*. OR = 1.42 [1.12–1.80] in the present analysis). Such findings reinforce the call for long-term monitoring of fatigue symptoms in irradiated CCSs, considering radiation dose optimization in treatment planning. Irradiation effects were site-specific, with head-and-neck exposure showing the strongest association with SF. This is biologically plausible: in a recent meta-analysis, head-and-neck radiotherapy in cancer survivors was consistently linked to fatigue, and brain/central structure dose has emerged as a determinant of fatigue trajectories [38]. Pediatric dose-mapping after cranial radiotherapy further supported a dose-brain network mechanism, with higher doses to basal ganglia and thalamo-cortical circuits correlating with impaired quality of life and cognition, health status subsequently inducing chronic and severe fatigue [39]. These findings emphasize minimizing integral brain dose where feasible and prioritizing fatigue screening, especially in CCSs with head-and-neck irradiation.

In contrast, no significant associations were found between fatigue and exposure to chemotherapy or the social deprivation index, suggesting that the contribution of these factors to long-term fatigue after treatment may be less direct or potentially mediated through other variables, such as female sex, younger age at diagnosis, as demonstrated in interaction factors tested in our study.

Compared to reference values derived from the general population, CCSs in the FCCSS consistently reported higher mean scores of “overall fatigue.” The comparison suggests a cancer-treatment-related excess and persistent fatigue among adult CCSs, as their general fatigue scores consistently exceed population norms derived from the general German population [22] and tended to intensify with increasing age after diagnosis. These findings should be interpreted with caution, as the reference norms used (from the German population) may not fully reflect the sociocultural and health characteristics of the French general population. Nevertheless, the magnitude and consistency of the differences observed support the interpretation of elevated fatigue in the FCCSS. Establishing French population-based normative data remains essential to improve the accuracy and clinical relevance of future comparisons.

### Strengths

Firstly, the present study, using the large, population-based FCCSS cohort, provided robust evidence of fatigue and SF among adult CCSs. Secondly, CNS tumor survivors and radiotherapy cancer treatment regimen, often underrepresented in other research work, were clearly identified by this study as having the highest fatigue burden. Thirdly, the use of the MFI-20 allowed for domain-specific fatigue profiling, revealing differential risk patterns. Lastly, comparison with age- and sex-specific general population norms highlights a persistent and cancer- and cancer-treatment-related excess fatigue.

### Limitations

This study presents some limitations. First, selection bias may have affected the generalizability of our results. The response rate was 41.3%, and participants differed from non-respondents in characteristics, including sex, age at the intention of fatigue questionnaire administration, and tumor type. However, the similarity in age at cancer diagnosis between responders and non-responders (i.e., deceased or loss to follow-up) partially mitigates this potential survivorship bias. Second, the cross-sectional nature of fatigue assessment did not allow us to confirm causal inference and limited ability to capture fatigue trajectories over time. Longitudinal data would be necessary to assess temporal changes related to aging and other late effects. Third, although the MFI-20 is a validated and widely used tool, no normative French reference values are currently available. We therefore relied on the German general population, which may not fully account for cross-cultural and health differences in the general population, as previously mentioned. Nevertheless, the large effect sizes and consistent trends across multiple fatigue dimensions and subgroups provide internal validity, supporting the robustness of our findings. Finally, some covariates, such as treatment intensity, cumulative doses, and detailed comorbidities, were not available in this analysis and may contribute to potential residual confounding. Fatigue may also reflect depression, and chronic inflammation-linked protein elevations seen in depression could explain fatigue. Indeed, a study found a 17% prevalence of moderate-to-severe depression among cancer survivors 5–10 years post-diagnosis [40]. While significant exposures, such as radiotherapy and chemotherapy, were included, future studies incorporating, for instance, biomarkers are needed to understand the mechanistic pathways underlying fatigue in CCSs, which will help manage this symptom more effectively in supportive care.

### Clinical implications

Our findings support that fatigue is a highly prevalent and disabling symptom among CCSs, affecting a broad spectrum of patients with different treatment modalities, age, or specific risk factors. While certain groups, such as women, older survivors, individuals with obesity, and CNS tumor survivors, may warrant particular attention, systematic screening should be implemented in routine long-term follow-up for all CCSs. Early identification must be followed by referral to dedicated supportive care interventions. Lifestyle promotion, particularly physical activity, should be systematically encouraged as the most promising intervention for prevention and management. This study underscores the need to consider fatigue as a frequent clinical issue that persists long-term after treatment, requiring structured management in CCSs, including the development and evaluation of targeted intervention programs. Clinical trials testing adapted physical activity interventions and supportive care strategies (such as nutritional and psychological support) hold promise for reducing fatigue and mitigating its long-term impact on quality of life and health outcomes in this population [41].

## Conclusion

Our large study contributes to a better understanding of fatigue as a prevalent, disabling, late effect in CCSs. We identified a significant burden of fatigue, with 16% of CCSs reporting SF. Female sex, age at diagnosis and survey, CNS tumor history, and radiotherapy were strong and independent predictors. These findings suggest the need for enhanced fatigue screening, along with targeted interventions and long-term supportive care, to identify high-risk subgroups for monitoring fatigue in CCSs, ultimately improving quality of life and health trajectories.

## Supplementary Information

Below is the link to the electronic supplementary material.

ESM 1 (47.5 KB DOCX)

## Data Availability

Data are accessible upon reasonable written request at florent.devathaire@gustaveroussy.fr (Gustave Roussy, EpiRad Team CESP Inser U1018, Paris-Saclay University), in compliance with strict institutional policies and the European General Data Protection Regulation (EU Regulation 2016/679).

## Declarations

### Clinical trial registration

The FCCS study is registered at ClinicalTrials.gov under the identifier NCT01620372.
