# Correction: Fatigue among adult survivors of childhood cancer: findings from the French Childhood Cancer Survivor Study

**DOI:** 10.1007/s11764-026-02043-z

**Published:** 2026-05-22

**Authors:** Médéa Locquet, Duc Hoan Vu, Rodrigue Allodji, Neige Journy, Chiraz El-Fayech, Charlotte Demoor-Goldschmidt, Laura Lenez, Cécile Thomas-Teinturier, Stéphanie Bolle, Nadia Haddy, Giao Vu-Bezin, Agnès Dumas, David Rajaonera, Nicolas Bougas, Jacques Grill, Christelle Dufour, Hélène Pacquement, Ibrahima Diallo, Jean Bernard Le-Provost, François Doz, Brice Fresneau, Florent de Vathaire

**Affiliations:** 1 Inserm Research Unit 1018 for Epidemiology and Health Population (CESP), Universite Paris-Saclay, UVSQ, Gustave Roussy, Villejuif, France; 2 University of Namur, Namur, Belgium; 3 Gustave Roussy, Villejuif, France; 4 Centro de Protonterapia, Grupo QuironSalud, Madrid, Spain; 5 IRD, ISSPAM, SESSTIM (Economic and Social Sciences of Health and Medical Information Processing), INSERM, Aix Marseille UnivCALIPSO Team, Marseille, France; 6 UMR 1123, Universite de Paris, ECEVE, INSERM (National Institute for Health and Medical Research), Paris, France; 7 Institut Curie, Paris, France; 8 Inserm Research Unit 1355 ‑ Molecular Radiotherapy and Innovative Therapeutics ‑ (RAMO‑IT), Universite Paris-Saclay, Gustave Roussy, Villejuif, France

**Correction: Journal of Cancer Survivorship**

**https://doi.org/10.1007/s11764-026-01995-6**

In this article the author’s name "Florent De Vathaire" was incorrectly written as "Florent Vathaire".

In Table 1, 2, 3 and 4 the numbers are all shifted upwards, For completeness and transparency, the correct versions are displayed below.

**Table 1:** Characteristics of participants from the French Childhood Cancer Survivors Study (FCCSS) cohort included in the present analysis (n=3,170)

Characteristics	Mean ± SD or n (%)
Sex	
*Women*	1,578 (49.8)
*Men*	1,592 (50.2)
Obesity	
*Yes*	245 (7.7)
*No*	2,925 (92.3)
Age at childhood cancer diagnosis (years)	6.1 ± 5.3
*0-1*	869 (27.4)
*2-4*	659 (20.8)
*5-8*	572 (18.0)
*9-12*	533 (16.8)
*13-20*	537 (17.0)
Age at questionnaire administration (years)	33.8 ± 8.7
*<30*	1,071 (33.8)
*30-39*	1,325 (41.8)
*40-49*	614 (19.4)
*≥50*	160 (5.0)
Year of cancer diagnosis	
*1945-1975*	732 (23.1)
*1976-1980*	569 (18.0)
*1981-1985*	700 (22.1)
*1986-1990*	438 (13.8)
*1991-1995*	369 (11.6)
*1996-2000*	362 (11.4)
First cancer type	
*Lymphomas*	577 (18.2)
*Central nervous system tumors*	333 (10.5)
*Neuroblastomas*	463 (14.6)
*Retinoblastomas*	142 (4.5)
*Renal tumors*	595 (18.8)
*Hepatic tumors*	34 (1.0)
*Malignant bone tumors*	297 (9.4)
*Soft-tissue tumors*	380 (12.0)
*Germ cells and gonadal tumors*	224 (7.1)
*Other carcinomas*	125 (3.9)
Post-treatment secondary pathologies	
*Second primary cancers*	337 (19.9)
*Cardiovascular complications*	138 (4.3)
*Cerebrovascular complications*	16 (0.5)
*Renal diseases*	74 (2.3)
*Type II diabetes mellitus*	58 (1.8)
*At least 1 incident secondary pathology*	270 (8.5)
Radiotherapy	
* Yes*	1,774 (56.0)
* No*	1,396 (44.0)
* Head-and-neck irradiation*	631 (19.9)
* Thoracic irradiation*	668 (21.1)
* Abdomen irradiation*	670 (21.1%)
* Pelvis irradiation*	137 (4.3)
* Leg and foot irradiation*	82 (2.6)
* Arm and hand irradiation*	7 (0.2)
Chemotherapy	
* Yes*	2,462 (77.7)
* No*	708 (22.3)
* Vinca alkaloids*	1,998 (63.0)
* Alkylation agents*	1,794 (56.6)
* Anthracyclines*	1,239 (39.1)
Social deprivation index	
*1*^*st*^* tertile*	989 (31.2)
*2*^*nd*^* tertile*	989 (31.2)
*3*^*rd*^* tertile*	989 (31.2)
*Not available*	203 (6.4)

**Table 2:** Multidimensional Fatigue Inventory (MFI-20) total and dimension scoring of the whole sample of childhood cancer survivors (CCSs), and according to sociodemographic and clinical characteristics (n=3,170)

	MFI-20 overall (/100) Mean±SD	General fatigue (/20) Mean±SD	Mental fatigue (/20) Mean±SD	Physical fatigue (/20) Mean±SD	Reduced activity (/20) Mean±SD	Reduced motivation (/20) Mean±SD
Score, *mean±standard deviation (SD)*	42.4±15.6	10.0±4.2	8.1±3.7	9.1±4.4	8.1±4.0	7.1±3.1
Sex						
*Women*	41.1±15.2	9.2±4.0	8.1±3.6	8.5±4.2	8.1±3.9	7.1±3.1
*Men*	43.7±15.9	10.7±4.2	8.0±3.8	9.7±4.4	8.2±4.1	7.2±3.2
*p*-value	<0.0001	<0.0001	0.46	<0.0001	0.59	0.58
Obesity						
*Yes*	48.5±17.4	11.3±4.3	9.3±4.2	10.9±4.7	9.0±4.4	8.1±3.6
*No*	41.7±15.2	9.8±4.1	8.0±3.6	8.9±4.3	7.9±8.2	7.0±3.1
*p*-value	<0.0001	<0.0001	<0.0001	<0.0001	0.002	<0.0001
Age at childhood cancer diagnosis (years)						
*0-1*	40.4±14.9	9.4±4.0	7.8±3.5	8.6±4.3	7.8±3.8	6.8±2.9
*2-4*	43.2±15.4	10.2±4.2	8.2±3.7	9.3±4.4	8.3±4.0	7.2±3.1
*5-8*	42.7±15.9	10.1±4.2	8.2±3.9	9.1±4.3	8.2±4.0	7.1±3.1
*9-12*	43.8±16.3	10.3±4.3	8.2±3.8	9.5±4.5	8.2±4.1	7.4±3.5
*13-20*	43.1±15.6	10.2±4.0	8.1±3.7	9.4±4.4	8.1±4.0	7.3±3.1
*p*-value	0.0002	<0.0001	0.215	0.0004	0.10	0.0004
administration (years)						
*<30*						
*30-39*						
*40-49*						
*≥50*						
*p*-value						
Age at questionnaire						
	40.5±14.3	9.3±3.9	8.0±3.5	8.3±4.0	8.1±3.8	6.7±2.9
	42.6±15.5	10.2±4.1	8.0±3.7	9.2±4.4	8.1±4.0	7.1±3.1
	44.3±16.8	10.6±4.3	8.2±3.9	10.0±4.6	8.1±4.2	7.5±3.3
	47.0±17.8	10.9±4.4	9.0±4.2	10.5±4.6	8.4±4.3	8.3±3.8
	<0.0001	<0.0001	0.02	<0.0001	0.85	<0.0001
Year of cancer diagnosis						
*1945-1975*	43.1±16.3	10.3±4.3	8.0±3.8	9.8±4.5	7.8±4.1	7.3±4.3
*1976-1980*	43.9±16.5	10.4±4.3	8.3±3.9	9.7±4.6	8.1±4.1	7.4±3.4
*1981-1985*	41.6±14.9	9.9±4.1	7.9±3.5	8.8±4.3	8.1±3.8	6.9±3.0
*1986-1990*	41.5±15.4	9.8±4.1	7.9±3.7	8.7±4.2	8.1±3.8	7.0±3.0
*1991-1995*	41.9±15.2	9.8±4.1	8.1±3.7	8.8±4.1	8.3±4.0	6.9±2.8
*1996-2000*	42.0±14.4	9.5±3.8	8.4±3.6	8.4±4.0	8.6±4.0	7.1±3.1
*p*-value	0.06	0.009	0.29	<0.0001	0.03	0.02
First cancer type						
*Lymphomas*	42.0±15.7	9.9±4.2	8.1±3.8	8.8±4.4	8.1±3.9	7.1±3.2
*Central nervous system tumors*	52.8±17.1	11.6±4.1	10.3±4.2	11.2±4.6	10.6±4.6	9.1±3.7
*Neuroblastomas*	40.8±14.9	9.6±4.1	7.8±3.6	8.7±4.3	8.0±3.9	6.8±2.8
*Retinoblastomas*	39.3±13.7	9.1±3.9	7.8±3.3	7.8±3.6	7.7±3.5	6.9±3.0
*Renal tumors*	41.1±14.6	10.0±4.1	7.8±3.6	9.1±4.3	7.5±3.6	6.7±3.0
*Hepatic tumors*	38.4±13.5	9.1±4.2	7.4±3.2	8.3±4.3	7.0±2.8	6.6±2.8
*Malignant bone tumors*	42.7±14.9	10.2±4.1	7.8±3.4	9.9±4.2	7.9±4.0	6.9±3.1
*Soft-tissue tumors*	40.4±14.5	9.6±4.1	7.6±3.3	8.7±4.2	7.7±3.8	6.8±2.9
*Germ cells and gonadal tumors*	40.2±14.7	9.5±4.2	7.8±3.3	8.1±4.1	7.8±3.9	6.9±2.7
*Other carcinomas*	43.5±16.0	10.5±4.1	8.2±3.7	9.0±4.2	8.3±4.0	7.5±3.2
*p*-value	<0.0001	<0.0001	<0.0001	<0.0001	<0.0001	<0.0001
Post-treatment secondary pathologies						
*Second primary cancers*	46.1±16.0	11.0±4.1	8.6±3.8	10.6±4.6	8.2±4.2	7.6±3.4
*Cardiovascular complications*	45.3±15.6	11.1±4.2	8.2±3.6	10.8±4.9	8.1±4.4	7.2±3.0
*Cerebrovascular complications*	53.2±17.3	12.1±4.5	10.5±3.4	11.7±4.0	9.8±4.1	9.1±4.0
*Renal diseases*	46.8±16.5	11.1±4.2	8.9±4.3	11.2±4.7	8.0±3.9	7.6±3.1
*Type II diabetes mellitus*	47.4±16.0	10.8±4.3	9.0±4.2	10.9±4.3	8.8±3.9	7.9±3.2
*At least 1 incident secondary pathology*	46.3±16.1	11.0±4.2	8.6±4.0	10.9±4.7	8.3±4.1	7.5±3.2
*p-value*	0.06	0.46	0.008	0.23	0.05	0.02
Radiotherapy						
*Yes*	44.2±15.1	10.3±4.2	8.4±3.8	9.8±4.5	8.3±4.1	7.3±3.3
*No*	39.7±14.2	9.4±4.0	7.6±3.4	8.2±4.0	7.8±3.8	6.7±2.8
*p*-value	<0.0001	<0.0001	<0.0001	<0.0001	0.005	<0.0001
Type of radiotherapy						
*Head-and-neck irradiation*	46.6±17.2	10.5±4.2	9.2±4.1	10.0±4.6	9.1±4.4	8.1±3.5
*Thoracic irradiation*	45.4±15.6	10.7±4.1	8.6±3.8	10.1±4.4	8.5±4.1	7.5±3.2
*Abdomen irradiation*	44.2±15.3	10.6±4.2	8.3±3.8	10.0±4.4	8.0±3.9	7.3±3.1
*Pelvis irradiation*	42.6±14.4	10.2±4.1	8.1±3.4	10.1±4.5	7.4±3.5	6.7±2.8
*Leg and foot irradiation*	43.1±15.4	10.3±4.1	8.0±3.5	10.0±4.3	7.5±3.9	7.3±3.4
*Arm and hand irradiation*	34.1±10.7	8.4±4.0	6.4±1.8	7.6±3.1	6.6±2.8	5.1±0.9
*p-value*	<0.0001	0.52	<0.0001	0.80	<0.0001	<0.0001
Chemotherapy						
*Yes*	41.8±15.0	9.8±4.1	8.0±3.6	9.0±4.3	8.0±3.9	6.9±3.0
*No*	43.6±16.9	10.2±4.8	8.4±3.6	9.3±4.4	8.4±4.2	7.4±3.4
*p*-value	<0.0001	0.05	0.02	0.20	0.03	0.0005
Type of chemotherapy agents						
*Vinca alkaloids*	41.6±14.6	9.8±4.1	7.9±3.6	8.9±4.3	8.0±3.8	7.0±3.0
*Alkylation agents*	42.1±15.2	9.8±4.2	8.1±3.7	9.0±4.3	8.1±3.9	7.0±3.1
*Anthracyclines*	41.4±14.8	9.8±4.1	7.9±3.6	8.9±4.3	7.9±3.8	6.9±3.0
*p-value*	0.39	0.99	0.17	0.73	0.36	0.60
Social deprivation index						
*1*^*st*^* tertile*	43.2±16.4	10.1±4.3	8.3±3.8	9.3±4.4	8.2±4.1	7.4±3.4
*2*^*nd*^* tertile*	42.0±15.2	10.0±4.1	8.0±3.6	9.1±4.4	8.1±3.9	7.0±3.0
*3*^*rd*^* tertile*	41.9±15.1	9.9±4.0	7.9±3.6	8.9±4.3	8.1±3.9	7.0±3.0
*Not available*	43.5±15.4	10.0±4.2	8.5±3.7	9.6±4.4	8.2±4.0	7.1±3.1
*p*-value	0.15	0.80	0.03	0.14	0.89	0.03

CI: confidence interval

**Table 3:** Associations between sociodemographic and clinical characteristics and MFI-20 fatigue score in childhood cancer survivors (CCSs) using multivariable linear regressions

	MFI-20 overall Adjusted^b^ β [95%CI]	General fatigue Adjusted β^b^ [95%CI]	Mental fatigue Adjusted^b^ β [95%CI]	Physical fatigue Adjusted^b^ β [95%CI]	Reduced activity Adjusted^b^ β [95%CI]	Reduced motivation Adjusted^b^ β [95%CI]
Sex						
*Men*	Reference	Reference	Reference	Reference	Reference	Reference
*Women*	1.06 [1.03-1.09]^a^	1.19 [1.15-1.23]^a^	0.98 [0.95-1.01]	1.15 [1.11-1.19]^a^	1.01 [0.98-1.03]	1.01 [0.98-1.03]
Obesity						
*No*	Reference	Reference	Reference	Reference	Reference	Reference
*Yes*	1.15 [1.07-1.25]^a^	1.17 [1.07-1.28]^a^	1.14 [1.04-1.25]^a^	1.27 [1.15-1.35]^a^	1.08 [1.00-1.21]	1.08 [1.00-1.17]^a^
Age at childhood cancer diagnosis (*years*)						
*0-1*	Reference	Reference	Reference	Reference	Reference	Reference
*2-4*	1.04 [1.00-1.08]^a^	1.05 [1.01-1.11]	1.03 [0.98-1.08]	1.04 [0.99-1.09]	1.04 [0.99-1.09]	1.04 [1.00-1.08]^a^
*5-8*	0.98 [0.94-1.03]	1.01 [0.96-1.06]	0.97 [0.92-1.03]	0.99 [0.93-1.05]	0.96 [0.90-1.02]	0.98 [0.94-1.03]
*9-12*	0.99 [0.94-1.04]	1.01 [0.95-1.07]	0.97 [0.91-1.03]	1.02 [0.95-1.09]	0.94 [0.88-1.01]	0.99 [0.94-1.04]
*13-20*	0.98 [0.92-1.03]	1.00 [0.93-1.07]	0.96 [0.90-1.03]	1.02 [0.95-1.12]	0.91 [0.83-0.98]	0.98 [0.92-1.03]
Age at questionnaire administration (*years*)						
*<30*	Reference	Reference	Reference	Reference	Reference	Reference
*30-39*	1.07 [1.04-1.13]^a^	1.13 [1.06-1.19]^a^	1.02 [0.97-1.07]	1.08 [1.03-1.16]^a^	1.07 [1.02-1.14]^a^	1.08 [1.04-1.16]^a^
*40-49*	1.16 [1.08-1.23]	0.19 [0.110.27]^a^	1.07 [1.00-1.16]	1.17 [1.08-1.30]^a^	1.15 [1.05-1.26]^a^	1.19 [1.12-1.88]^a^
*≥50*	1.23 [1.13-1.38]^a^	1.25 [1.09-1.42]^a^	0.16 [0.06-0.23]^a^	1.22 [1.07-1.40]^a^	1.25 [1.09-1.43]^a^	1.32 [1.19-1.52]^a^
Year of cancer diagnosis						
*1945-1975*	Reference	Reference	Reference	Reference	Reference	Reference
*1976-1980*	1.06 [1.01-1.12]^a^	1.05 [0.99-1.12]	1.06 [1.01-1.14]	1.03 [0.97-1.09]	1.09 [1.03-1.17]^a^	1.06 [1.01-1.13]^a^
*1981-1985*	1.05 [1.00-1.12]	1.05 [0.99-1.13]	1.04 [0.98-1.13]	0.97 [0.90-1.04]	1.15 [1.07-1.25]^a^	1.04 [0.98-1.12]
*1986-1990*	1.04 [0.98-1.09]	1.04 [0.97-1.13]	1.04 [0.97-1.13]	0.95 [0.89-1.03]	1.16 [1.06-1.26]^a^	1.05 [0.98-1.13]
*1991-1995*	1.05 [0.99-1.14]	1.04 [0.96-1.14]	1.08 [1.00-1.19]	0.96 [0.89-1.05]	1.21 [1.08-1.32]^a^	1.07 [0.99-1.16]
*1996-2000*	1.09 [1.02-1.19]^a^	1.05 [0.96-1.16]	1.13 [1.03-1.25]^a^	0.95 [0.87-1.05]	1.31 [1.17-1.51]^a^	1.13 [1.02-1.23]^a^
First cancer type						
*Lymphomas*	Reference	Reference	Reference	Reference	Reference	Reference
*Central nervous system tumors*	1.30 [1.23-1.38]^a^	1.19 [1.12-1.27]^a^	1.31 [1.25-1.42]^a^	1.35 [1.25-1.46]^a^	1.38 [1.25-1.52]^a^	1.32 [1.23-1.42]^a^
*Neuroblastomas*	0.99 [0.94-1.04]	0.99 [0.92-1.05]	0.98 [0.92-1.05]	1.00 [0.93-1.07]	1.00 [0.93-1.06]	0.99 [0.93-1.05]
*Retinoblastomas*	0.96 [0.89-1.03]	0.94 [0.85-1.03]	0.97 [0.89-1.06]	0.93 [0.83-1.02]	0.97 [0.87-1.06]	1.00 [0.92-1.07]
*Renal tumors*	0.98 [0.93-1.03]	1.01 [0.95-1.06]	0.96 [0.91-1.02]	1.02 [0.96-1.08]	0.94 [0.88-1.00]	0.95 [0.90-1.00]
*Hepatic tumors*	0.96 [0.84-1.08]	0.95 [0.80-1.12]	0.94 [0.79-1.09]	1.00 [0.81-1.20]	0.88 [0.73-1.06]	1.00 [0.86-1.16]
*Malignant bone tumors*	1.02 [0.97-1.07]	1.02 [0.96-1.08]	0.98 [0.92-1.04]	1.15 [1.06-1.23]a	0.98 [0.91-1.04]	0.96 [0.91-1.02]
*Soft-tissue tumors*	0.96 [0.91-1.01]	0.96 [0.90-1.01]	0.96 [0.90-1.02]	0.98 [0.92-1.04]	0.95 [0.89-1.01]	0.96 [0.91-1.01]
*Germ cells and gonadal tumors*	0.96 [0.90-1.01]	0.94 [0.87-1.01]	0.99 [0.92-1.06]	0.90 [0.83-0.97]^a^	0.98 [0.91-1.04]	0.99 [0.93-1.06]
*Other carcinoma*	1.02 [0.95-1.09]	1.02 [0.94-1.13]	1.02 [0.93-1.12]	1.00 [0.90-1.09]	1.04 [0.95-1.15]	1.04 [0.97-1.14]
Post-treatment secondary pathologies						
*None*	Reference	Reference	Reference	Reference	Reference	Reference
*Second primary cancers*	1.08 [1.04-1.13]^a^	1.08 [1.05-1.15]^a^	1.05 [0.94-1.17]^a^	1.10 [1.01-1.22]^a^	1.27 [0.91-1.60]	0.97 [0.92-1.08]^a^
*Cardiovascular complications*	1.07 [1.01-1.14]^a^	1.09 [1.04-1.15]^a^	1.12 [1.05-1.17]^a^	1.10 [0.99-1.23]	1.22 [0.97-1.36]	1.26 [1.00-1.58]
*Cerebrovascular complications*	1.23 [1.03-1.47]^a^	1.13 [1.04-1.21]^a^	1.08 [1.04-1.14]^a^	1.07 [1.01-1.13]^a^	1.16 [0.98-1.26]	1.02 [0.92-1.14]
*Renal diseases*	1.12 [1.02-1.20]^a^	1.20 [0.97-1.47]	1.03 [0.96-1.10]	1.14 [1.08-1.20]^a^	1.18 [0.96-1.26]	1.09 [0.99-1.22]
*Type II diabetes mellitus*	1.10 [1.01-1.21]^a^	1.12 [1.08-1.23]^a^	1.34 [1.08-1.66]^a^	1.18 [1.08-1.28]^a^	1.02 [0.98-1.08]	1.07 [1.01-1.12]^a^
*At least 1 incident secondary pathology*	1.08 [1.05-1.15]^a^	1.12 [1.05-1.17]^a^	1.07 [1.05-1.13]^a^	1.19 [1.13-1.26]^a^	1.04 [0.99-1.11]	1.07 [1.01-1.13]^a^
Radiotherapy						
*No*	Reference	Reference	Reference	Reference	Reference	Reference
*Yes*	1.06 [1.03-1.09]	1.05 [1.02-1.09]	1.07 [1.04-1.13]	1.13 [1.07-1.32]	1.02 [0.98-1.65]	1.04 [1.01-1.07]
Type of radiotherapy						
*None*	Reference	Reference	Reference	Reference	Reference	Reference
*Head-and-neck irradiation*	1.11 [1.08-1.15]^a^	0.81 [0.62-1.06]	1.02 [0.93-1.12]	1.02 [0.95-1.09]	1.12 [1.07-1.16]^a^	1.06 [1.02-1.10]^a^
*Thoracic irradiation*	1.08 [1.05-1.13]^a^	1.07 [1.03-1.10]^a^	0.82 [0.60-1.13]	0.99 [0.89-1.09]	1.08 [1.01-1.17]^a^	1.01 [0.97-1.05]
*Abdomen irradiation*	1.06 [1.03-1.09]^a^	1.08 [1.05-1.13]^a^	1.16 [1.12-1.19]^a^	0.85 [0.62-1.17]	1.07 [0.98-1.20]	0.93 [0.86-1.01]
*Pelvis irradiation*	1.01 [0.95-1.06]	1.07 [1.03-1.12]^a^	1.09 [1.05-1.13]^a^	1.10 [1.05-1.15]^a^	0.84 [0.77-1.20]	0.94 [0.85-1.04]
*Leg and foot irradiation*	1.01 [0.94-1.10]	1.01 [0.95-1.09]	1.05 [1.01-1.09]^a^	1.14 [1.09-1.18]^a^	1.15 [1.10-1.20]^a^	1.15 [1.12-1.20]^a^
*Arm and hand irradiation*	1.02 [0.97-1.04]	0.99 [0.94-1.03]	0.91 [0.86-1.11]	0.97 [0.88-1.10]	1.01 [0.94-1.14]	0.96 [0.87-1.05]
Chemotherapy						
*No*	Reference	Reference	Reference	Reference	Reference	Reference
*Yes*	1.03 [0.99-1.06]	1.02 [0.98-1.06]	1.01 [0.97-1.04]	1.04 [0.99-1.08]	1.02 [0.98-1.07]	1.03 [0.99-1.06]
Type of chemotherapy agents						
*None*	Reference	Reference	Reference	Reference	Reference	Reference
*Vinca alkaloids*	0.99 [0.97-1.01]	0.99 [0.97-1.03]	0.98 [0.95-1.01]	0.99 [0.96-1.03]	0.98 [0.94-1.01]	1.01 [0.98-1.03]
*Alkylation agents*	1.01 [0.99-1.03]	0.99 [0.97-1.02]	1.01 [0.98-1.03]	1.01 [0.98-1.04]	1.01 [0.98-1.05]	0.97 [0.94-0.99]
*Anthracyclines*	0.99 [0.96-1.02]	1.01 [0.97-1.03]	0.97 [0.94-0.99]	1.02 [0.98-1.05]	0.96 [0.93-0.99]	0.95 [0.91-0.99]
Social deprivation index						
*1*^*st*^* tertile*	Reference	Reference	Reference	Reference	Reference	Reference
*2*^*nd*^* tertile*	0.98 [0.95-1.01]	0.99 [0.95-1.03]	0.97 [0.93-1.03]	0.98 [0.94-1.02]	0.99 [0.95-1.03]	0.96 [0.92-1.00]^a^
*3*^*rd*^* tertile*	0.99 [0.96-1.02]	1.00 [0.96-1.04]	0.97 [0.93-1.00]	0.98 [0.94-1.03]	1.00 [0.96-1.04]	0.97 [0.94-1.01]
*Not available*	1.02 [0.97-1.08]	1.01 [0.94-1.07]	1.03 [0.96-1.09]	1.05 [0.98-1.13]	1.02 [0.95-1.09]	0.99 [0.93-0.95]

Abbreviations: CI: confidence interval

^a^*p*<0.05

^b^Models adjusted for sex, obesity, age at childhood cancer diagnosis, age at questionnaire administration, year of cancer diagnosis, first cancer type, post-treatment secondary pathologies, radiotherapy, type of radiotherapy, chemotherapy, type of chemotherapy agents, and social deprivation index

**Table 4:** Associations between sociodemographic and clinical characteristics and the reporting of severe fatigue in CCSs of the FCCSS cohort

Characteristics	Adjusted^a^ OR [95%CI]
Sex	
*Men (reference)*	1.00
*Women*	1.28 [1.03-1.56]
Obesity	
*No (reference)*	1.00
*Yes*	2.40 [1.37-3.59]
Age at childhood cancer diagnosis (years)	
*0-1 (reference)*	1.00
*2-4*	1.16 [0.85-1.60]
*5-8*	0.86 [0.60-1.24]
*9-12*	0.81 [0.54-1.22]
*13-20*	0.67 [0.42-1.06]
Age at questionnaire administration (years)	
*<30 (reference)*	1.00
*30-39*	1.72 [1.23-2.40]
*40-49*	3.07 [1.91-4.93]
*≥50*	4.40 [2.27-8.54]
Year of cancer diagnosis	
*1945-1975 (reference)*	1.00
*1976-1980*	1.50 [1.06-2.12]
*1981-1985*	1.33 [0.88-2.01]
*1986-1990*	1.52 [0.96-2.42]
*1991-1995*	1.69 [0.98-2.90]
*1996-2000*	1.85 [1.02-3.36]
First cancer type	
*Lymphomas (reference)*	1.00
*Central nervous system tumors*	3.05 [2.10-4.43]
*Neuroblastomas*	1.13 [0.73-1.73]
*Retinoblastomas*	0.62 [0.31-1.23]
*Renal tumors*	0.83 [0.56-1.22]
*Hepatic tumors*	1.21 [0.40-3.65]
*Malignant bone tumors*	1.14 [0.76-1.72]
*Soft-tissue tumors*	0.94 [0.63-1.39]
*Germ cells and gonadal tumors*	1.02 [0.63-1.64]
*Other carcinomas*	1.53 [0.89-2.66]
Post-treatment secondary pathologies	
*None (reference)*	1.00
*Second primary cancers*	1.30 [0.97-1.71]
*Cardiovascular complications*	0.65 [0.35-1.22]
*Cerebrovascular complications*	2.00 [0.56-6.77]
*Renal diseases*	1.47 [0.74-2.83]
*Type II diabetes mellitus*	1.11 [0.78-2.22]
*At least 1 incident secondary pathology*	1.62 [1.18-2.21]
Radiotherapy	
*No (reference)*	1.00
*Yes*	1.42 [1.12–1.80]
Type of radiotherapy	
*None (reference)*	1.00
*Head-and-neck irradiation*	2.11 [1.69-2.62]
*Thoracic irradiation*	1.34 [1.06-1.69]
*Abdomen irradiation*	1.16 [0.91-1.47]
*Pelvis irradiation*	0.77 [0.44-1.26]
*Leg and foot irradiation*	1.16 [0.61-2.05]
*Arm and hand irradiation*	0.30 [0.09-1.89]
Chemotherapy	
*No (reference)*	1.00
*Yes*	1.10 [0.84-1.44]
Type of chemotherapy agents	
*None (reference)*	1.00
*Vinca alkaloids*	0.85 [0.69-1.05]
*Alkylation agents*	1.06 [0.84-1.34]
*Anthracyclines*	0.96 [0.75-1.22]
Social deprivation index	
*1*^*st*^* tertile (reference) (least deprived)*	1.00
*2*^*nd*^* tertile*	0.80 [0.62-1.02]
*3*^*rd*^* tertile (most deprived)*	0.81 [0.63-1.04]
*Not available*	1.02 [0.68-1.54]

Abbreviations: CCS: childhood cancer survivor; FCCSS: French Childhood Cancer

Survivors Study; OR: odds ratio; CI: confidence interval

^a^ Models adjusted for sex, obesity, age at childhood cancer diagnosis, age at questionnaire administration, year of cancer diagnosis, first cancer type, post-treatment secondary pathologies, radiotherapy, type of radiotherapy, chemotherapy, type of chemotherapy agents, and social deprivation index

Correct version of Table 1

Correct version of Table 2

Correct version of Table 3

Correct version of Table 4

The original article has been corrected.

