# Supplementary material for: Fatigue among adult survivors of childhood cancer: findings from the French Childhood Cancer Survivor Study
Source: J Cancer Surviv. 2026 Mar 14;20(Suppl 2):1674–88. doi: 10.1007/s11764-026-01995-6 (PMC13744513; doi:10.1007/s11764-026-01995-6)
Supplement: Supplementary file 1 [file 11764_2026_1995_MOESM1_ESM.docx]

Supplementary Materials

| Supplementary Table 1. Characteristics of patients included versus excluded from the present French Childhood Cancer Survivors Study (FCCSS) cohort analysis. | | | | |
| --- | --- | --- | --- | --- |
|  | Dead before questionnaire delivery (n=1,218) | Refusal or loss of follow-up or missing data (n=3,282) | Included (n=3,170) | *p*-value* |
| Gender |  |  |  |  |
| *Male* | 694 | 1,929 (54%) | 1 592 (46%) | <0.0001 |
| *Female* | 524 | 1 353 (45%) | 1 578 (55%) |  |
| Age at childhood cancer diagnosis (years) | | | | |
| *0-1* | 212 | 1051 (55%) | 869 (45%) |  |
| *2-4* | 275 | 665 (50%) | 659 (50%) |  |
| *5 - 8* | 213 | 422 (49%) | 572 (51%) | 0.001 |
| *9- 12* | 292 | 598 (47%) | 533 (53%) |  |
| *13-20* | 226 | 546 (50%) | 537 (50%) |  |
| Year of diagnosis (years) | | | | |
| *1948-1975* | 441 | 230 (24%) | 732 (76%) |  |
| *1976-1980* | 199 | 261 (31%) | 569 (69%) |  |
| *1981-1985* | 182 | 364 (34%) | 700 (66%) | <0.0001 |
| *1986-1990* | 166 | 648 (60%) | 438 (40%) |  |
| *1991-1995* | 131 | 813 (69%) | 369 (31%) |  |
| *1996-2000* | 99 | 966 (73%) | 362 (27%) |  |
| Index of social deprivation FDEP09 (tertile) | | | | |
| *1* | 329 | 965 (49%) | 989 (51%) |  |
| *2* | 273 | 847 (46%) | 989 (54%) |  |
| *3* | 418 | 1118 (53%) | 989 (47%) | 0.7 |
| No available | 198 | 352 (63%) | 203 (37%) |  |
| First cancer type (ICCC groups) | | | | |
| *Lymphoma* | 167 | 534 (48%) | 577 (52%) |  |
| *CNS tumors* | 398 | 409 (55%) | 333 (45%) |  |
| *Peripheral nervous tumor* | 102 | 469 (50%) | 463 (50%) |  |
| *Retinoblastomas* | 55 | 422 (75%) | 142 (25%) |  |
| *Renal tumors* | 133 | 412 (41%) | 595 (59%) |  |
| *Hepatic tumors* | 9 | 36 (51%) | 34 (49%) |  |
| *Bone sarcomas* | 125 | 264 (47%) | 297 (53%) | <0.0001 |
| *Soft-tissue sarcomas* | 129 | 350 (48%) | 380 (52%) |  |
| *Germ cells and gonadal tumors* | 42 | 203 (48%) | 224 (52%) |  |
| *Other carcinomas* | 57 | 181 (59%) | 125 (41%) |  |
| Treatment |  |  |  |  |
| *Radiotherapy* | 995 | 1,427 (44%) | 1,774 (56%) | <0.0001 |
| *Chemotherapy* | 883 | 2,330 (49%) | 2,462 (51%) | <0.01 |

*p*-value of the comparison between the refusal patients and the included ones. Trend test for age at diagnosis, year of diagnosis, and index of social deprivation, and heterogeneity test for sex, type of cancer, radiotherapy, and chemotherapy.

| **Supplementary Table 2**. Associations between sociodemographic and clinical characteristics and MFI-20 fatigue score in childhood cancer survivors (CCSs) using multivariable linear regressions on log-transformed values | | | | | | |
| --- | --- | --- | --- | --- | --- | --- |
|  | MFI-20 overall  Adjusted^b^ β [95%CI] | General fatigue  Adjusted β [95%CI] | Mental fatigue  Adjusted β [95%CI] | Physical fatigue  Adjusted β [95%CI] | Reduced activity  Adjusted β [95%CI] | Reduced motivation  Adjusted β [95%CI] |
| Sex  *Men*  *Women* | Reference  0.06 [0.03-0.09]^a^ | Reference  0.17[0.14-0.21]b | Reference  -0.02 [-0.05-0.01] | Reference  0.14 [0.10-0.17]^a^ | Reference  0.01 [-0.02-0.03] | Reference  0.01 [-0.02-0.03] |
| Obesity  *No*  *Yes* | Reference  0.14 [0.07-0.22]^a^ | Reference  0.16 [0.07-0.25]^a^ | Reference  0.13 [0.04-0.22]^a^ | Reference  0.24 [0.14-0.30]^a^ | Reference  0.08 [-0.00-0.19] | Reference  0.08 [0.00-0.16]^a^ |
| Age at childhood cancer diagnosis (*years*)  *0-1*  *2-4*  *5-8*  *9-12*  *13-20* | Reference  0.04 [0.00-0.08]^a^  -0.02 [-0.06-0.03]  -0.01 [-0.06-0.04]  -0.02 [-0.08-0.03] | Reference  0.05 [0.01-0.10]  0.01 [-0.04-0.06]  0.01 [-0.05-0.07]  0.00 [-0.07-0.07] | Reference  0.03 [-0.02-0.08]  -0.03 [-0.08-0.03]  -0.03 [-0.09-0.03]  -0.04 [-0.11-0.03] | Reference  0.04 [-0.01-0.09]  -0.01 [-0.07-0.05]  0.02 [-0.05-0.09]  0.02 [-0.05-0.11] | Reference  0.04 [-0.01-0.09]  -0.04 [-0.10-0.02]  -0.06 [-0.13-0.01]  -0.09 [-0.19- -0.02] | Reference  0.04 [0.00-0.08]^a^  -0.02 [-0.06-0.03]  -0.01 [-0.06-0.04]  -0.02 [-0.08-0.03] |
| Age at questionnaire administration (*years*)  *<30*  *30-39*  *40-49*  *≥50* | Reference  0.07 [0.04-0.12]^a^  0.15 [0.08-0.21]  0.21 [0.12-0.32]^a^ | Reference  0.12 [0.06-0.17]^b^  0.19 [0.110.27]^b^  0.222 [0.09-0.35]^b^ | Reference  0.02 [-0.03-0.07]  0.07 [-0.00-0.15]  0.16 [0.06-0.231^a^ | Reference  0.08 [0.03-0.15]^a^  0.16 [0.08-0.26]^a^  0.20 [0.07-0.34]^a^ | Reference  0.07 [0.02-0.13]^a^  0.14 [0.05-0.23]^a^  0.22 [0.09-0.36]^a^ | Reference  0.08 [0.04-0.15]^a^  0.17 [0.11-0.63]^a^  0.28 [0.17-0.42]^a^ |
| Year of cancer diagnosis  *1945-1975*  *1976-1980*  *1981-1985*  *1986-1990*  *1991-1995*  *1996-2000* | Reference  0.06 [0.01-0.11]^a^  0.05 [-0.00-0.11]  0.04 [-0.02-0.09]  0.05 [-0.01-0.13]  0.09 [0.02-0.17]^a^ | Reference  0.05[-0.01-0.11]  0.05[-0.01-0.12]  0.04[-0.03-0.12]  0.04[-0.04-0.13]  0.05[-0.04-0.15] | Reference  0.06 [0.01-0.13]  0.04 [-0.02-0.12]  0.04 [-0.03-0.12]  0.08 [-0.00-0.17]  0.12 [0.03-0.22]^a^ | Reference  0.03[-0.03-0.09]  -0.03[-0.10-0.04]  -0.05[-0.12-0.03]  -0.04[-0.12-0.05]  -0.05[-0.14-0.05] | Reference  0.09 [0.03-0.16]^a^  0.14 [0.07-0.22]^a^  0.15 [0.06-0.23]^a^  0.19 [0.08-0.28]^a^  0.27 [0.16-0.41]^a^ | Reference  0.06 [0.01-0.12]^a^  0.04 [-0.02-0.11]  0.05 [-0.02-0.12]  0.07 [-0.01-0.15]  0.12 [0.02-0.21]^a^ |
| First cancer type  *Lymphomas*  *Central nervous system tumors*  *Neuroblastomas*  *Retinoblastomas*  *Renal tumors*  *Hepatic tumors*  *Malignant bone tumors*  *Soft-tissue tumors*  *Germ cells and gonadal tumors*  *Other carcinomas* | Reference  0.26 [0.21-0.32]^a^  -0.01 [-0.06-0.04]  -0.04 [-0.12-0.03]  -0.02 [-0.07-0.03]  -0.04 [-0.17-0.08]  0.02 [-0.03-0.07]  -0.04 [-0.09-0.01]  -0.04 [-0.11-0.01]  0.02 [-0.05-0.09] | Reference  0.17 [0.11-0.24]^a^  -0.01[-0.08-0.05]  -0.06[-0.16-0.03]  0.01[-0.05-0.06]  -0.05[-0.22-0.11]  0.02[-0.04-0.08]  -0.04[-0.11-0.01]  -0.06[-0.14-0.01]  0.02[-0.06-0.12] | Reference  0.27 [0.22-0.35]^a^  -0.02 [-0.08-0.05]  -0.03 [-0.12-0.06]  -0.04 [-0.09-0.02]  -0.06 [-0.23-0.09]  -0.02 [-0.08-0.04]  -0.04 [-0.11-0.02]  -0.01 [-0.08-0.06]  0.02 [-0.07-0.11] | Reference  0.30 [0.22-0.38]^a^  0.00 [-0.07-0.07]  -0.07 [-0.19-0.02]  0.02 [-0.04-0.08]  -0.00 [-0.21-0.18]  0.14 [0.06-0.21]^a^  -0.02 [-0.08-0.04]  -0.10 [-0.19- -0.03]^a^  -0.00 [-0.10-0.09] | Reference  0.32 [0.22-0.42]^a^  -0.00 [-0.07-0.06]  -0.03 [-0.14-0.06]  -0.06 [-0.13-0.00]  -0.13 [-0.31-0.06]  -0.02 [-0.09-0.04]  -0.05 [-0.12-0.01]  -0.02 [-0.09-0.04]  0.04 [-0.05-0.14] | Reference  0.28 [0.21-0.35]^a^  -0.01 [-0.07-0.05]  -0.00 [-0.08-0.07]  -0.05 [-0.11-0.00]  0.00 [-0.15-0.15]  -0.04 [-0.09-0.02]  -0.04 [-0.09-0.01]  -0.01 [-0.07-0.06]  0.04 [-0.03-0.13] |
| Post-treatment incident secondary pathologies  *None*  *Second primary cancers*  *Cardiovascular complications*  *Cerebrovascular complications*  *Renal diseases*  *Type II diabetes mellitus*  *At least 1 incident secondary pathology* | Reference  0.08 [0.04-0.12]^a^  0.07 [0.01-0.13]^a^  0.21 [0.03-0.39]^a^  0.11 [0.02-0.18]^a^  0.10 [0.01-0.19]^a^  0.08 [0.05-0.14]^a^ | Reference  0.09 [0.04-0.14]^a^  0.12 [0.04-0.19]^a^  0.18 [-0.03-0.39]  0.11 [0.08-0.21]^a^  0.05 [-0.06-0.16]  0.11 [0.05-0.16]^a^ | Reference  0.08 [0.04-0.13]^a^  0.03 [-0.04-0.10]  0.29 [0.08-0.51]^a^  0.10 [0.01-0.20]^a^  0.10 [-0.01-0.21]  0.07 [0.01-0.12]^a^ | Reference  0.13 [0.08-0.18]^a^  0.17 [0.08-0.25]^a^  0.24 [0.01-0.47]^a^  0.20 [0.09-0.31]^a^  0.15 [0.03-0.28]^a^  0.17 [0.12-0.23]^a^ | Reference  0.02 [-0.02-0.08]  -0.03 [-0.08-0.08]  0.23 [0.00-0.46]  0.02 [-0.08-0.13]  0.12 [0.00-0.25]  0.04 [-0.01-0.10] | Reference  0.07 [0.02-0.11]^a^  0.02 [-0.04-0.09]  0.19 [-0.01-0.39]  0.08 [-0.01-0.18]  0.09 [-0.01-0.20]  0.07 [0.01-0.12] |
| Radiotherapy  *No*  *Yes* | Reference  0.06 [0.03-0.09] | Reference  0.05 [0.02-0.09] | Reference  0.07 [0.04-0.12] | Reference  0.12 [0.07-0.28] | Reference  0.02[-0.02-0.5) | Reference  0.04 [0.01-0.07] |
| Type of radiotherapy  *None*  *Head-and-neck irradiation*  *Thoracic irradiation*  *Abdomen irradiation*  *Pelvis irradiation*  *Leg and foot irradiation*  *Arm and hand irradiation* | Reference  0.11 [0.08-0.14]^a^  0.08 [0.05-0.12]^a^  0.06 [0.03-0.09]^a^  0.01 [-0.05-0.06]  0.01 [-0.06-0.10]  -0.21 [-0.47-0.06] | Reference  0.07 [0.03-0.10]^a^  0.08 [0.05-0.12]^a^  0.07 [0.03-0.11]^a^  0.01 [-0.05-0.09]  0.02 [-0.07-0.12]  -0.20 [-0.51-0.12] | Reference  0.15[0.11-0.18]^a^  0.09 [0.05-0.12]^a^  0.05 [0.01-0.09]^a^  0.02 [-0.05-0.09]  -0.01 [-0.11-0.9]  -0.16 [-0.48-0.16] | Reference  0.10 [0.05-0.14]^a^  0.13 [0.09-0.17]^a^  0.11 [0.07-0.15]^a^  0.08 [0.01-0.16]^a^  0.07 [-0.02-0.18]  -0.17 [-0.25-0.18] | Reference  0.14 [0.10-0.18]^a^  0.06 [0.02-0.10]^a^  0.01 [-0.03-0.05]  -0.07 [--.015-0.01]  -0.06 [-0.16-0.04]  -0.17 [-0.51-0.17] | Reference  0.14 [0.11-0.18]^a^  0.05 [0.02-0.09]^a^  0.04 [0.01-0.07]^a^  -0.06 [-0.13-0.01]  0.01 [-0.09-0.09]  -0.26 [-0.56-0.03] |
| Chemotherapy  *No*  *Yes* | Reference  0.03 [-0.01-0.06] | Reference  0.02 [-0.02-0.06] | Reference  0.01[-0.03-0.04] | Reference  0.04 [-0.01-0.08] | Reference  0.02 [-0.02-0.07] | Reference  0.03 [-0.01-0.06] |
| Type of chemotherapy agents  *None*  *Vinca alkaloids*  *Alkylation agents*  *Anthracyclines* | Reference  -0.01 [-0.03-0.01]  0.01 [-0.01-0.03]  -0.01 [-0.04-0.2] | Reference  -0.01 [-0.03-0.03]  -0.01 [-0.03-0.02]  0.01 [-0.03-0.03] | Reference  -0.02 [-0.05-0.01]  0.01 [-0.02-0.03]  -0.03 [-0.06-0.01] | Reference  -0.01 [-0.04-0.03]  0.01 [-0.02-0.04]  0.02 [-0.02-0.05] | Reference  -0.02 [-0.06-0.01]  0.01 [-0.02-0.05]  -0.04 [-0.07-0.01] | Reference  -0.01 [-0.04-0.01]  0.01 [-0.02-0.03]  -0.03 [-0.06-0.01] |
| Social deprivation index  *1^st^ tertile*  *2^nd^ tertile*  *3^rd^ tertile*  *Not available* | Reference  -0.02 [-0.05-0.01]  -0.01 [-0.04-0.02]  0.02 [-0.03-0.08] | Reference  -0.01 [-0.05-0.03]  0.00 [-0.04-0.04]  0.01 [-0.06-0.07] | Reference  -0.03 [-0.07-0.03]  -0.03 [-0.07-0.00]  0.03 [-0.04-0.09] | Reference  -0.02 [-0.06-0.02]  -0.02 [-0.06-0.03]  0.05 [-0.02-0.12] | Reference  -0.01 [-0.05-0.03]  -0.00 [-0.04-0.04]  0.02 [-0.05-0.09] | Reference  -0.04 [-0.08- -0.00]^a^  -0.03 [-0.06-0.01]  -0.01 [-0.07- -0.05] |

Abbreviations: CI: confidence interval

^a^*p*<0.05

^b^ Models adjusted for sex, obesity, age at childhood cancer diagnosis, age at questionnaire administration, year of cancer diagnosis, first cancer type, post-treatment secondary pathologies, radiotherapy, type of radiotherapy, chemotherapy, type of chemotherapy agents, and social deprivation index

|  | **Supplementary Table 3**. Associations between sociodemographic and clinical characteristics and the reporting of severe fatigue in **central nervous system cancer survivors** of the FCCSS cohort | |
| --- | --- | --- |
|  | Characteristics | Adjusted OR [95%CI] |
|  | Sex  *Men (reference)*  *Women* | 1.00  1.39[0.85-2.27] |
|  | Obesity  *No (reference)*  *Yes* | 1.00  2.31 [0.86-6.24] |
|  | Age at childhood cancer diagnosis (years)  *0-1 (reference)*  *2-4*  *5-8*  *9-12*  *13-20* | 1.00  0.82[0.32-2.06]]  1.10[0.43-2.76]  0.78[0.29-2.11]  0.77[0.24-2.50] |
|  | Age at questionnaire administration (years)  *<30 (reference)*  *30-39*  *40-49*  *≥50* | 1.00 0.75[0.35-1.63]  1.79[0.53-6.09]  5.37[0.90-32.19] |
|  | Year of cancer diagnosis  *1945-1975 (reference)*  *1976-1980*  *1981-1985*  *1986-1990*  *1991-1995*  *1996-2000* | 1.00  1.29[0.45-3.68]  1.35[0.45-4.04]  1.46[0.45-4.79]  0.91[0.26-3.64]  1.11[0.25-4.81] |
|  | Post-treatment secondary pathologies  *None (reference)*  *Second primary cancers*  *Cardiovascular complications*  *Cerebrovascular complications*  *Renal diseases*  *Type II diabetes mellitus*  *At least 1 incident secondary pathology* | 1.00  0.97 [0.51-1.81]  1.55 [0.94-1.98]  1.79 [0.62-2.26]  1.70 [0.99-2.01]  0.99 [0.89-1.10]  1.99 [0.58-1.68] |
|  | Radiotherapy  *No (reference)*  *Yes* | 1.00   1.38 [0.57–3.34] |
|  | Type of radiotherapy  *None (reference)*  *Head-and-neck irradiation*  *Thoracic irradiation*  *Abdomen irradiation*  *Pelvis irradiation*  *Leg and foot irradiation*  *Arm and hand irradiation* | 1.00  1.60 [0.78-3.50]  0.84 [0.47-1.47]  0.63 [0.33-1.17]  1.69 [0.31-8.19]  2.00 [0.23-3.24]  Not applicable |
|  | Chemotherapy  *No (reference)*  *Yes* | 1.00  1.05 [0.61-1.81] |
| Type of chemotherapy agents  *None (reference)*  *Vinca alkaloids*  *Alkylation agents*  *Anthracyclines* | | 1.00  0.70 [0.43-1.15]  0.91 [0.40-2.05]  1.03 [0.23-2.31] |
|  | Social deprivation index  *1^st^ tertile (reference)*  *2^nd^ tertile*  *3^rd^ tertile*  *Not available* | 1.00  0.75 [0.41-1.38]  0.60 [0.32-1.12]  0.90 [0.27-2.96] |

Abbreviations: FCCSS: French Childhood Cancer Survivors Study; OR: odds ratio; CI: confidence interval

^a^ Models adjusted for sex, obesity, age at childhood cancer diagnosis, age at questionnaire administration, year of cancer diagnosis, first cancer type, post-treatment secondary pathologies, radiotherapy, type of radiotherapy, chemotherapy, type of chemotherapy agents, and social deprivation index

|  | **Supplementary Table 4**. Associations between sociodemographic and clinical characteristics and the reporting of severe fatigue in **non**-**central nervous system cancer survivors** of the FCCSS cohort | |
| --- | --- | --- |
|  | Characteristics | Adjusted^a^ OR [95%CI] |
|  | Sex  *Men (reference)*  *Women* | 1.00  1.28 [1.02-1.61] |
|  | Obesity  *No (reference)*  *Yes* | 1.00  2.40 [1.38-4.20] |
|  | Age at childhood cancer diagnosis (years)  *0-1 (reference)*  *2-4*  *5-8*  *9-12*  *13-20* | 1.00  1.23 [0.87-1.73]  0.77 [0.51-1.17]  0.80 [0.51-1.27]  0.60 [0.36-1.01] |
|  | Age at questionnaire administration (years)  *<30 (reference)*  *30-39*  *40-49*  *≥50* | 1.00  2.09 [1.43-3.04]  3.58 [2.12-6.05]  4.52 [2.16-9.45] |
|  | Year of cancer diagnosis  *1945-1975 (reference)*  *1976-1980*  *1981-1985*  *1986-1990*  *1991-1995*  *1996-2000* | 1.00  1.60 [1.11-2.32]  1.37 [0.87-2.16]  1.65 [0.99-2.76]  1.96 [1.08-3.56]  2.06 [1.05-4.04] |
|  | Post-treatment secondary pathologies  *None (reference)*  *Second primary cancers*  *Cardiovascular complications*  *Cerebrovascular complications*  *Renal diseases*  *Type II diabetes mellitus*  *At least 1 incident secondary pathology (reference)* | 1.00  1.37 [0.99-1.87]  0.70 [0.36-1.35]  1.49 [0.27-2.55]  1.58 [0.80-3.10]  1.04 [0.47-2.21]  1.67 [1.19-2.31] |
|  | Radiotherapy  *No (reference)*  *Yes* | 1.00  1.43 [1.11-1.84] |
|  | Type of radiotherapy  *None (reference)*  *Head-and-neck irradiation*  *Thoracic irradiation*  *Abdomen irradiation*  *Pelvis irradiation*  *Leg and foot irradiation*  *Arm and hand irradiation* | 1.00  1.32 [0.96-1.79]  1.41 [0.89-3.39]  1.23 [0.93-1.62]  0.70 [0.61-1.24]  1.04 [0.52-1.91]  2.48 [0.44-2.15] |
|  | Chemotherapy  *No (reference)*  *Yes* | 1.00  1.14 [0.82-1.57] |
| Type of chemotherapy agents  *None (reference)*  *Vinca alkaloids*  *Alkylation agents*  *Anthracyclines* | | 1.00  0.99 [0.79-1.27]  1.18 [0.91-1.54]  1.15 [0.88-1.48] |
|  | Social deprivation index  *1^st^ tertile (reference)*  *2^nd^ tertile*  *3^rd^ tertile*  *Not available* | 1.00  0.79 [0.60-1.05]  0.86 [0.65-1.14]  1.07 [0.69-1.66] |

Abbreviations: FCCSS: French Childhood Cancer Survivors Study; OR: odds ratio; CI: confidence interval

^a^ Models adjusted for sex, obesity, age at childhood cancer diagnosis, age at questionnaire administration, year of cancer diagnosis, first cancer type, post-treatment secondary pathologies, radiotherapy, type of radiotherapy, chemotherapy, type of chemotherapy agents, and social deprivation index

| **Supplementary Table 5**. Associations between radiation dose received to the brain during radiation therapy and the reporting of severe fatigue in CCSs of the FCCSS cohort | |
| --- | --- |
| Characteristics | Adjusted^a^ OR [95%CI] |
| Mean radiation dose received to whole brain during radiation therapy (*Gray* [Gy])  *No (reference)*  *≤ 1 Gy*  *>1-5 Gy*  *>5-20 Gy*  *>20-40 Gy*  *>40Gy* | 1.00   1.26 [0.97-1.64]  2.07 [1.41-3.00]  2.03 [1.39-2.98]  3.32 [2.33-4.72]  3.99 [2.11-7.56] |

^a^ Models adjusted for sex, obesity, age at childhood cancer diagnosis, age at questionnaire administration, year of cancer diagnosis, first cancer type, post-treatment secondary pathologies, radiotherapy, type of radiotherapy, chemotherapy, type of chemotherapy agents, and social deprivation index
